# Photoluminescence and afterglow of Dy^3+^ doped CaAl_2_O_4_ derived *via* sol–gel combustion†

**DOI:** 10.1039/d2ra05008k

**Published:** 2022-11-08

**Authors:** Bao-gai Zhai, Meng Meng Chen, Yuan Ming Huang

**Affiliations:** School of Microelectronics and Control Engineering, Changzhou University Changzhou 213164 China dongshanisland@126.com

## Abstract

With doping concentration varying from 0.1 to 5.0 mol%, a series of Dy^3+^ doped calcium aluminate (CaAl_2_O_4_:Dy^3+^) phosphors were synthesized *via* a sol–gel combustion technique. The phase, morphology, photoluminescence (PL), afterglow, and thermoluminescence (TL) glow curves of CaAl_2_O_4_:Dy^3+^ were investigated by means of X-ray diffractometry, scanning electron microscopy, transmission electron microscopy, PL spectroscopy, afterglow spectroscopy, and TL dosimetry, respectively. It is found that: (i) oxygen vacancies and Dy^3+^ work as two independent sets of luminescence centers of PL for CaAl_2_O_4_:Dy^3+^; (ii) Dy^3+^ works as the luminescence center of afterglow for CaAl_2_O_4_:Dy^3+^; (iii) the afterglow of CaAl_2_O_4_:Dy^3+^ lasts for about 115 min at the optimal doping concentration of around 0.8 mol%; and (iv) multiple traps, which are sensitive to doping concentration, are present in CaAl_2_O_4_:Dy^3+^. The PL and afterglow mechanisms of CaAl_2_O_4_:Dy^3+^ are discussed to reveal the processes of charged carrier excitation, migration, trapping, detrapping, and radiative recombination in CaAl_2_O_4_:Dy^3+^.

## Abstract 2

A systematic investigation into the photoluminescence and afterglow mechanisms of trivalent Dy ion doped CaAl_2_O_4_.

## Graphical abstract

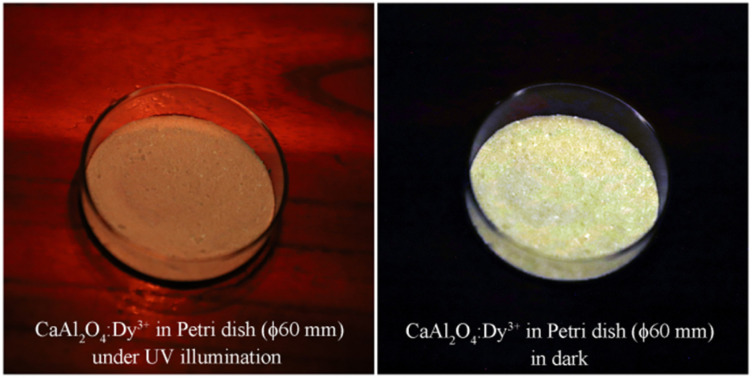

## 1. Introduction

Rare-earth doped calcium aluminate phosphors have been intensively studied due to their excellent afterglow properties,^1–4^ among which Dy^3+^ doped calcium aluminate (CaAl_2_O_4_:Dy^3+^) is a well-known inorganic phosphor which emits white photoluminescence (PL) under ultraviolet excitation.^5^ Apart from its white PL, CaAl_2_O_4_:Dy^3+^ exhibits a white afterglow even after the removal of ultraviolet excitation.^6^ As reported by Liu *et al.* in 2005, Dy^3+^ works as the luminescence center of afterglow for CaAl_2_O_4_:Dy^3+^ and the white afterglow of solid state reaction derived CaAl_2_O_4_:Dy^3+^ lasts 32 min at the optimal doping concentration of 2 at%.^6^ For afterglow materials, the processes of charged carrier excitation, migration, trapping, detrapping, and radiative recombination are critically important to understand their afterglow properties.^7–9^ For example, long afterglow can be achieved at room temperature only when traps have an appropriate activation energy somewhere around 0.65 eV whereas shallow traps (*E* ≤ 0.4 eV) and deep traps (*E* > 2 eV) are not favorable because they can be emptied either easily or with difficulty at room temperature.^7^ Up to date, only a single report exists on the afterglow of CaAl_2_O_4_:Dy^3+^, leaving the processes of charged carrier excitation, migration, trapping and detrapping not fully revealed. The lack of such knowledge hampers the comprehensive understanding of the afterglow mechanism of CaAl_2_O_4_:Dy^3+^.

With respect to solid state reaction derived CaAl_2_O_4_:Dy^3+^, sol–gel combustion derived CaAl_2_O_4_:Dy^3+^ is abundant in oxygen and calcium vacancies. In CaAl_2_O_4_:Dy^3+^, oxygen vacancies are potential electron traps because they are positively charged whereas calcium vacancies are potential hole traps because they are negatively charged.^8,10^ Therefore, sol–gel combustion derived CaAl_2_O_4_:Dy^3+^ phosphors are suitable for exploring the afterglow mechanisms of CaAl_2_O_4_:Dy^3+^. In this paper, we report the PL and afterglow properties of sol–gel combustion derived CaAl_2_O_4_:Dy^3+^ with the doping concentrations varying from 0.1 to 5.0 mol%. A picture on the PL and afterglow mechanisms of CaAl_2_O_4_:Dy^3+^ is given to reveal the processes of charged carriers' excitation, migration, trapping, detrapping, and recombination in CaAl_2_O_4_:Dy^3+^.

## 2. Experimental section

### 2.1 Sol–gel combustion synthesis of CaAl_2_O_4_:Dy^3+^

CaAl_2_O_4_:Dy^3+^ phosphors with the doping concentrations varying from 0.1 to 5.0 mol% were synthesized *via* the sol–gel combustion with urea as fuel.^11–13^ All reagents were in analytical grade and provided by Sinopharm Chemical Reagents Co., Ltd (Shanghai, China). The purity of dysprosium oxide (Dy_2_O_3_) was 99.99%. Under stirring with a magnetic bar, Ca(NO_3_)_2_·4H_2_O (0.2 mol), Al(NO_3_)_3_·9H_2_O (0.4 mol), urea (6.0 mol), H_3_BO_3_ (0.02 mol) and stoichiometric amount of Dy_2_O_3_ were dissolved in deionized water (600 mL) to form a transparent solution. A homogeneous solution was obtained after the mixture was stirred vigorously for 60 min in a glass beaker. After having been aged at room temperature for two weeks, the solution was ready for the sol–gel combustion. Urea and boric acid functioned as the fuel and flux, respectively. Alumina crucibles, each with the volume capacity of 50 mL, were employed as the reaction containers. Half filled with the aged solution, the solution-containing alumina crucible was transferred into an air-filled box furnace for self-propagating combustion. The temperature in the furnace was preset at 780 °C. After the solution-containing crucible was transferred into the furnace, the temperature in the furnace was gradually dropped to about 706 °C. Heated at such high temperatures, water in the solution got boiling and the starting materials (fuels and metal nitrates) in the crucible were partially decomposed until the organic fuels were automatically ignited to initiate the exothermic reactions. The sol–gel combustion yielded voluminous gases and bright flames. In this work, the synthesis was initiated by point-heating of a small part of the mixture in the crucible, and the ignition was started at around 750 °C. Once started, a wave of exothermic reactions swept through the remaining material in the crucible. This combustion synthesis lasted for about 40 s, which was exceptionally fast when compared to high-temperature solid state reactions. During the sol–gel combustion, a large amount of energy was released from the exothermic reactions, which in turn raised the temperature in the furnace up to 830 °C. Measured with an infrared thermometer, the temperature in the flame was up to 1300 °C. After the fire was extinguished, the crucible was taken out of the furnace immediately. The total holding time of the crucible in the furnace was about 4 min. White powders were resulted after the sol–gel combustion. According to molar ratio of Dy^3+^ to Ca^2+^ in the starting materials, a series of CaAl_2_O_4_:Dy^3+^ phosphors were obtained with the nominal doping concentration of Dy^3+^ varying in the range of 0.1–5.0 mol%. No further calcination was applied to the sol–gel combustion derived CaAl_2_O_4_:Dy^3+^.

### 2.2 Solid state synthesis of CaAl_2_O_4_:Dy^3+^ phosphors

For comparison, solid state reaction derived CaAl_2_O_4_:Dy^3+^ phosphors were synthesized with CaCO_3_ (0.02 mol), Al_2_O_3_ (0.02 mol), H_3_BO_3_ (0.002 mol), Dy_2_O_3_ as raw materials. Stoichiometric mixtures of the raw materials were homogeneously mixed and ground. Subsequently the mixture was transferred into alumina crucibles and sintered at 900 °C for 4 h in an air-filled electric tube furnace. The sintered products were ground again in an agate mortar, then the powder products were calcined at 1350 °C for 10 h in air. According to the molar ratio of Dy^3+^ to Ca^2+^ in the starting materials, the doping concentration of Dy^3+^ in the solid state reaction derived CaAl_2_O_4_:Dy^3+^ varied in the range 0.1–10.0 mol%.

### 2.3 Phase, morphology and elemental composition of CaAl_2_O_4_:Dy^3+^ phosphors

X-Ray diffraction (XRD) profiles of CaAl_2_O_4_:Dy^3+^ phosphors were recorded on X-ray diffractometer (D/max 2500 PC, Rigaku Corporation, Akishima, Japan) using Cu Kα radiation (*λ* = 0.15405 nm). The scanning electron microscope (SEM) (model S-4800, Hitachi, Tokyo, Japan) was employed to analyze the morphology of the synthesized products. The SEM was coupled with a silicon drifted detector as the X-ray analyzer to record the energy dispersive X-ray (EDX) spectrum of the synthesized products. The micrographs of CaAl_2_O_4_:Dy^3+^ nanocrystals were recorded on a transmission electron microscope (TEM) (model JEOL JEM-2100, Japan Electronics Corp, Akishima, Japan). Samples for TEM analysis were prepared by suspending the particles in ethanol under the excitation of ultrasonification and then drying a drop of the suspension on a carbon-coated copper grid.

### 2.4 PL excitation and emission spectra of CaAl_2_O_4_:Dy^3+^

The PL excitation spectrum of CaAl_2_O_4_:Dy^3+^ was measured with the fluorescence spectrometer F-7000 (Hitachi, Japan). The spectrophotometer (Tianjin Gangdong Ltd., Tianjin, China) was used to acquire the steady-state PL spectra of CaAl_2_O_4_:Dy^3+^. The excitation source of the PL spectrum was provided by a helium–cadmium laser (Kimmon Electric Co. Ltd., Tokyo, Japan). The emission wavelength and the output power of the laser radiation were 325 nm and 13 mW, respectively.

### 2.5 Afterglow spectra and thermoluminescence (TL) glow curves of CaAl_2_O_4_:Dy^3+^

The afterglow spectra of CaAl_2_O_4_:Dy^3+^ were recorded with the PL spectrophotometer (Tianjin Gangdong Ltd, Tianjin, China) immediately after the ultraviolet irradiation of a high-pressure mercury lamp was blocked off. The output power of the high-pressure mercury lamp was 175 W. The irradiation duration of the high-pressure lamp irradiation was 3 min. Afterglow decay curve was taken by focusing the afterglows into the entrance slit of the spectrometer. The TL glow curves of CaAl_2_O_4_:Dy^3+^ were measured on a TL meter constructed according to the scheme given by Yamashita *et al.*^14^ The phosphor was placed on an electrically heated plate, the temperature of the plate was controlled with a program in computer. As the temperature of the plate was raised linearly with time, the light output of the phosphor was recorded using a photomultiplier in a photon-counting mode. A bialkali photomultiplier tube (model H10425, Hamamatsu, Japan) was used in the TL meter for the luminescence detection, it covers the 350–600 nm wavelength range. Prior to the TL measurements, CaAl_2_O_4_:Dy^3+^ phosphors were exposed to the 254 nm irradiation of a low-pressure mercury lamp for 5 min. The output power of the low-pressure mercury lamp was 32 W. The TL signals of CaAl_2_O_4_:Dy^3+^ phosphors were recorded when the CaAl_2_O_4_:Dy^3+^ phosphors were heated from 10 to 200 °C at a rate of 2 °C s^−1^.

## 3. Results and discussions

### 3.1 Phase and morphology of CaAl_2_O_4_:Dy^3+^

**Fig. 1:**
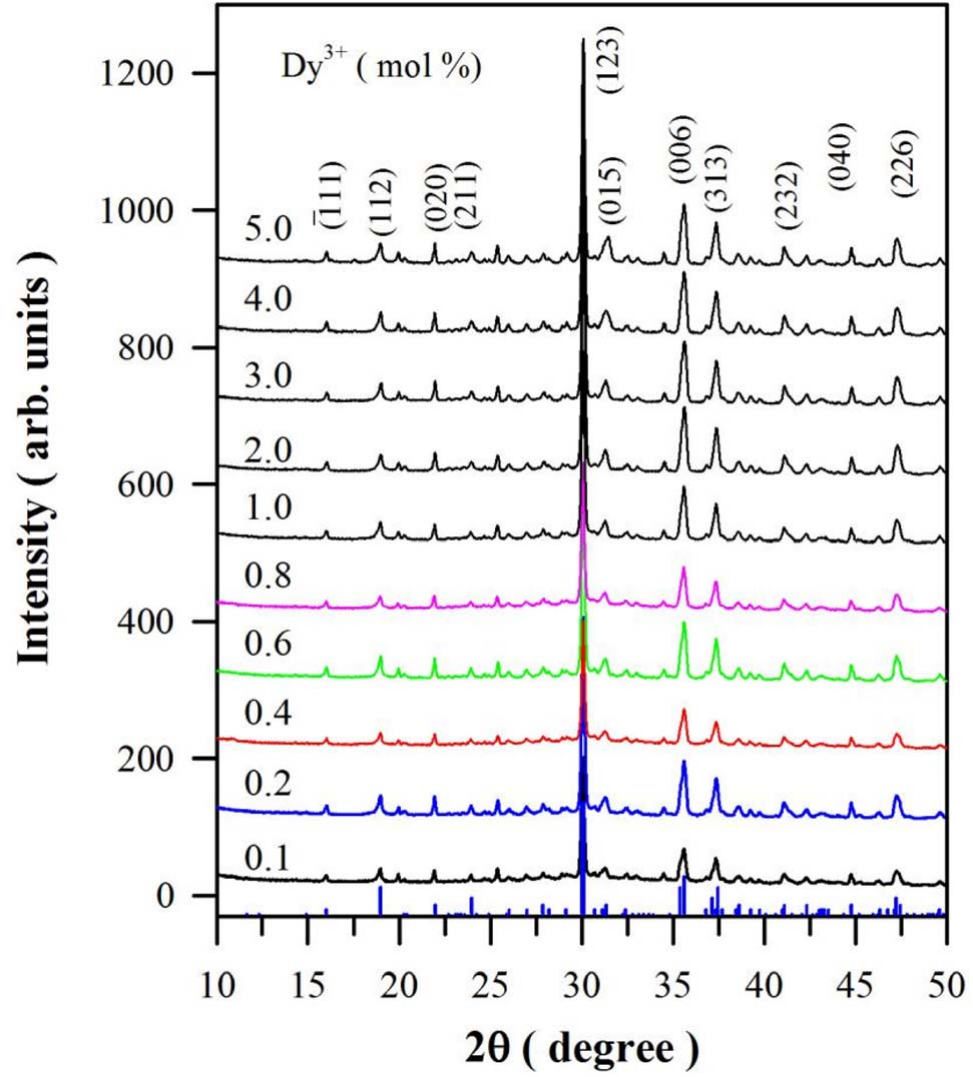
XRD curves of CaAl_2_O_4_:Dy^3+^ with doping concentrations varying from 0.1 to 5.0 mol%. The standard diffraction data of CaAl_2_O_4_ (JCPDS 23-1036) are displayed at the bottom of this figure for comparison.

Fig. 1 represents the XRD profiles of CaAl_2_O_4_:Dy^3+^ with different doping concentrations. The XRD data of monoclinic CaAl_2_O_4_ registered in Joint Committee on Powder Diffraction Standards (JCPDS) card no. 23-1306 are depicted at the bottom of the figure for comparison. The scale of the intensity of powder X-ray diffraction is counts of photons received by the detector. For the purpose of clarity, the XRD curves in Fig. 1 are evenly spaced after having been shifted upwards one by one, so the scale of the intensity of powder XRD in Fig. 1 is arbitrary units. As can be seen in Fig. 1, each XRD profile of CaAl_2_O_4_:Dy^3+^ exhibits distinct peaks at 2*θ* = 16.01, 18.99, 21.98, 23.97, 30.08, 31.35, 35.63, 37.46, 41.11, 44.76 and 47.24°. According to the diffraction data registered in JCPDS card no. 23-1036, these peaks can be attributed to the X-ray diffractions from crystallographic planes (1̄11), (112), (020), (211), (123), (015), (006), (313), (232), (040) and (226) of monoclinic CaAl_2_O_4_, respectively. Among these peaks, the strongest one is located at 30.08° and indexed as (123). Actually, this peak is contributed jointly by two crystallographic planes (2̄20) and (123) of monoclinic CaAl_2_O_4_, which are located at 2*θ* = 30.11 and 30.05°, respectively. Being closely packed and nearly equal in diffraction intensity, the two peaks are not distinguishable in the XRD profiles. This is the reason why some researchers indexed the peak at around 30.8° as (220).^7,8^ Obviously, the diffraction peaks in Fig. 1 match well with the standard XRD diffractograms of monoclinic CaAl_2_O_4_.^3,15,16^ Furthermore, these peaks are found to agree well with the calculated XRD diffractograms of monoclinic CaAl_2_O_4_ (Fig. S1†). The absence of a secondary phase in the XRD profiles indicates that Dy^3+^ ions are successfully incorporated into CaAl_2_O_4_ without introducing new phase in the crystal structure when doping concentration is lower than 5.0 mol%. For sol–gel combustion, nitrate forms of starting materials are highly necessary. Instead of Dy_2_O_3_, for example, Dy(NO_3_)_3_ is favored as the starting material. It is known that Dy_2_O_3_ can be dissolved into dilute HNO_3_ solution to form Dy (NO_3_)_3_.^9^ In this work, Dy_2_O_3_ is used as a starting material to provide the source of Dy^3+^ because a tiny amount of Dy_2_O_3_ can be dissolved into the aqueous solution of Al(NO_3_)_3_ and urea after stirring with a magnetic bar for a couple of hours at a temperature higher than 20 °C. Since the synthesis process occurs at high temperatures generated by the self-propagating combustion, this method is ideally suited for the production of refractory materials including ceramics CaAl_2_O_4_.

Monoclinic CaAl_2_O_4_ is known to have the stuffed tridymite structure with the space group *P*2_1_/*n* and *Z* = 12. Fig. S2(a)† displays the conventional unit cell of monoclinic CaAl_2_O_4_. Taken from the Inorganic Crystal Structure Database (ICSD), the lattice parameters of monoclinic CaAl_2_O_4_ (*a* = 0.8694 nm, *b* = 0.8093 nm, *c* = 1.5210 nm, and *β* = 90.1665°) are used to construct the crystal structure in present work (ICSD #159925). Obviously, this unit cell consists of 12 Ca sites, 24 Al sites and 48 O sites. As labeled as sites Ca1, Ca2 and Ca3 in Fig. S2(a),† there are three crystallographically different sites for Ca^2+^ in the unit cell of CaAl_2_O_4_. Two of the Ca sites are six-coordinated and one Ca site is nine-coordinated.^17^ In monoclinic CaAl_2_O_4_, a three-dimensional network is formed by corner-sharing [AlO_4_] tetrahedra, the Ca^2+^ ions are located along the channels in *b*-direction. Fig. S2(b)† illustrates the schematic view of the monoclinic CaAl_2_O_4_ along the *b*-direction. Such a crystal structure makes it easy for Dy^3+^ ions to substitute the Ca^2+^ sites in CaAl_2_O_4_. The effective ionic radii of cations depend on their coordination numbers (CN). In the case of site occupancy, the effective ionic radii of Ca^2+^ are 0.100 nm (CN = 6) and 0.118 nm (CN = 9), the effective ionic radii of Dy^3+^ are 0.091 nm (CN = 6) and 0.108 nm (CN = 9).^18^ Due to the comparable ionic sizes, Dy^3+^ ions tend to replace the Ca^2+^ ions in the lattice of CaAl_2_O_4_.

**Fig. 2:**
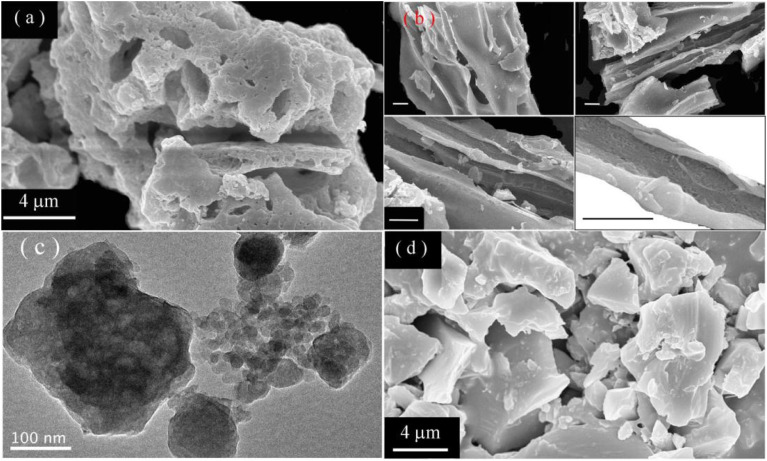
(a) SEM micrograph of sol–gel combustion derived CaAl_2_O_4_:Dy^3+^ to show aggregates; (b) SEM micrographs of sol–gel combustion derived CaAl_2_O_4_:Dy^3+^ to display the channels formed in the aggregates, each scale bar represents 2 μm; (c) TEM micrograph of sol–gel combustion derived CaAl_2_O_4_:Dy^3+^ display the nanocrystals formed in the aggregates; (d) SEM micrograph of solid state reaction derived CaAl_2_O_4_:Dy^3+^ (5.0 mol%) at 1350 °C for 10 h.

Fig. 2(a) displays the SEM micrograph of sol–gel combustion derived CaAl_2_O_4_:Dy^3+^ (5.0 mol%). As shown in Fig. 2(a), the sol–gel combustion derived CaAl_2_O_4_:Dy^3+^ phosphors are in the form of irregular aggregates with their dimensions up to 20 μm. Sol–gel combustion derived aluminate nanocrystals are prone to forming large aggregates. Micrometer-sized pores are randomly distributed in the aggregates due to the production of a large amount of gases during the reaction.^3,8,11–13,19,20^Fig. 2(b) shows the SEM micrographs of CaAl_2_O_4_:Dy^3+^ to display the channels formed in the aggregates, each scale bar represents 2 μm. It is evident that channels with different diameters are present in the aggregate. Fig. 2(c) shows the TEM micrograph of the CaAl_2_O_4_:Dy^3+^. Obviously, each aggregate consists of a large number of nanocrystals. Previous work has evidenced that one aggregate consists of a number of nanocrystals whose sizes vary from 10 nm to 80 nm.^4,16,17,21^ As a contrast, solid state reaction derived CaAl_2_O_4_:Dy^3+^ exhibits quite different morphology. Fig. 2(d) shows the SEM micrograph of solid state reaction derived CaAl_2_O_4_:Dy^3+^ (5.0 mol%) at 1350 °C for 10 h. As shown in Fig. 2(d), CaAl_2_O_4_:Dy^3+^ phosphors are in the form of irregular blocks without any porous channels. The dimension of the blocks is as large as 10 μm in diameter. A comparison of the micrographs reveals that the sol–gel combustion derived CaAl_2_O_4_:Dy^3+^ has large surface area, which in turn renders the phosphor rich in oxygen vacancies and abundant in traps for charged carriers. Till date, several rare earth doped CaAl_2_O_4_ materials are studied and available in literature. For example, Liu *et al.* prepared CaAl_2_O_4_:Dy^3+^*via* solid state reaction at 1350 °C.^6^ Due to the features of solid state reaction, the solid state reaction derived CaAl_2_O_4_:Dy^3+^ phosphors are different from the sol–gel combustion derived ones in the aspects of population density of intrinsic defects. Especially in the sol–gel combustion method, addition of H_3_BO_3_ lowers reaction temperature, accelerates a diffusion of raw materials, and eventually influences the afterglow properties. Detailed discussions are given by Takeuchi and Kishine in the case of Eu^2+^ and Dy^3+^ codoped SrAl_2_O_4_.^22^

### 3.2 EDX spectrum of CaAl_2_O_4_:Dy^3+^

**Fig. 3:**
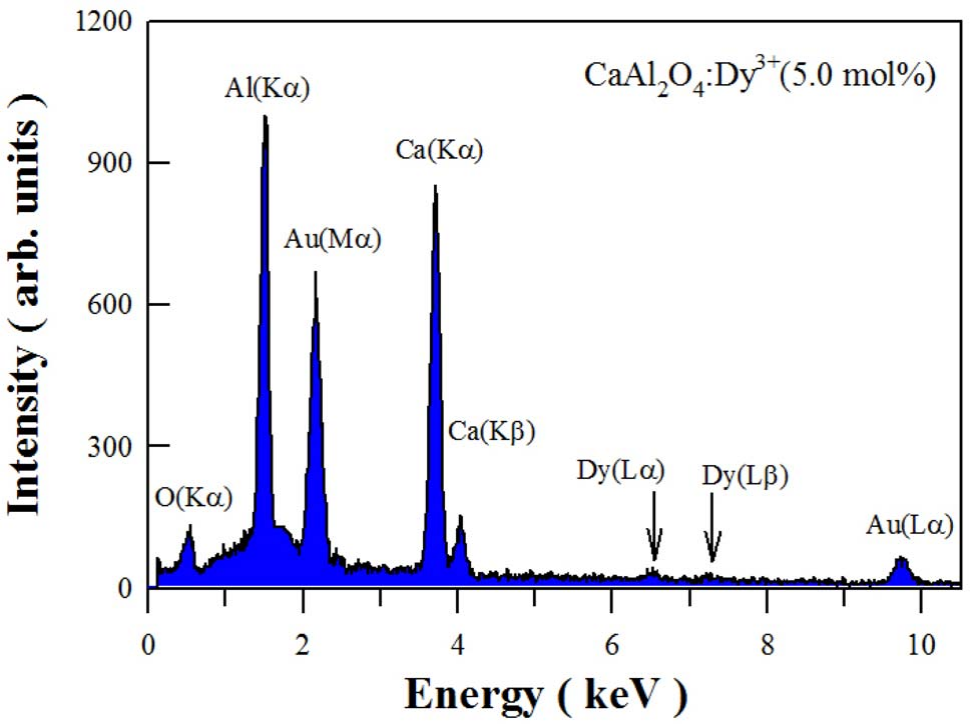
EDX spectrum of sol–gel combustion derived CaAl_2_O_4_:Dy^3+^ (5.0 mol%).

Fig. 3 depicts the EDX spectrum of sol–gel combustion derived CaAl_2_O_4_:Dy^3+^ (5.0 mol%). As can be seen in Fig. 3, the characteristic X-ray emissions of O(Kα), Al(Kα) and Ca(Kα_2_), and Ca(Kβ_1,3_) are located at 0.525, 1.486, 3.693, and 4.013 keV, respectively. Moreover, the characteristic emissions of Dy(Lα_1_) and Dy(Lβ_1_) can be identified in the EDX spectrum at 6.495 and 7.248 keV, respectively. The X-ray emissions of Au(Mα_1_) at 2.122 keV and Au(Lα_1_) at 9.713 keV are due to Au sputtering for the convenience of SEM characterization. Apparently, the EDX spectrum of CaAl_2_O_4_:Dy^3+^ confirms the presence of elements Ca, Al, O and Dy in the sample.

### 3.3 XPS spectrum of CaAl_2_O_4_:Dy^3+^

**Fig. 4:**
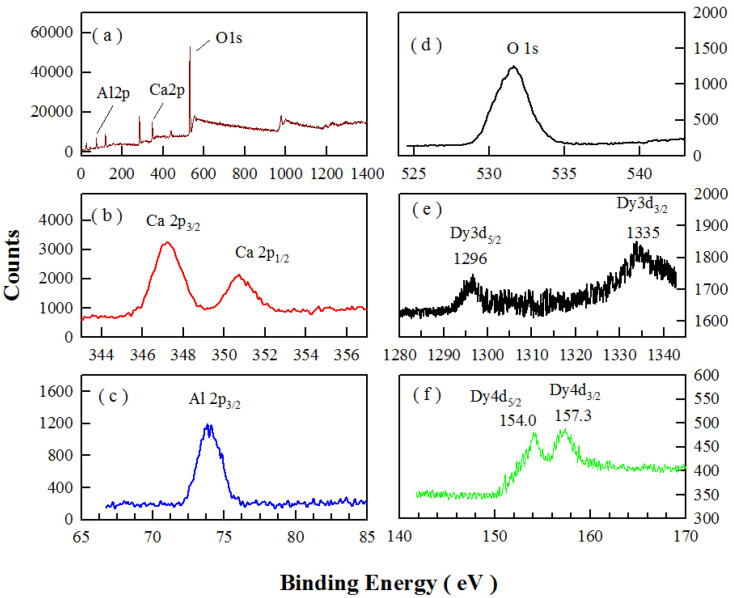
XPS survey scan (a) and high-resolution XPS spectra of Ca 2p (b), Al 2p (c), O 1s (d), Dy 3d (e), and Dy 4d (f) in sol–gel combustion derived CaAl_2_O_4_:Dy^3+^ (2.0 mol%).

The oxidation states of chemical elements in CaAl_2_O_4_:Dy^3+^ are investigated with the XPS. Fig. 4 represents the XPS survey scan (a) and high-resolution XPS spectra of Ca 2p (b), Al 2p (c), O 1s (d), Dy 3d (e), and Dy 4d (f) in sol–gel combustion derived CaAl_2_O_4_:Dy^3+^. The nominal doping concentration of Dy^3+^ is 2.0 mol%. As can be seen in Fig. 4(b–d), the binding energies of Ca 2p_3/2_, Ca 2p_1/2_, Al 2p_1/2_, and O 1s are located at approximately 347.3, 350.9, 74.1, and 531.8 eV, respectively. Fig. 4(e) shows that the binding energies of Dy 3d_5/2_ and Dy 3d_3/2_ are located at around 1297.6 and 1335.1 eV, respectively. Fig. 4(f) shows that the binding energies of Dy 4d_5/2_ and Dy 4d_3/2_ are located at around 154.0 and 157.3 eV, respectively. The data in Fig. 4 point out the presence of Ca, Al, O and Dy^3+^ in the phosphor.

### 3.4 PL excitation spectrum of CaAl_2_O_4_:Dy^3+^

**Fig. 5:**
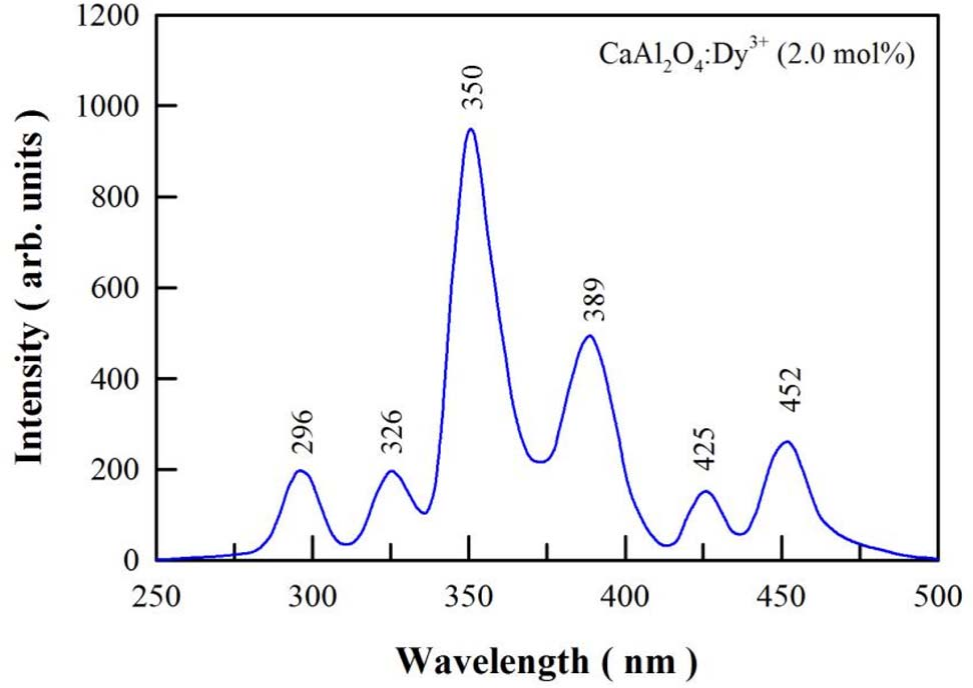
PL excitation spectrum of sol–gel combustion derived CaAl_2_O_4_:Dy^3+^ (2.0 mol%). The emission wavelength is fixed at 574 nm.

Fig. 5 shows the PL excitation spectrum of sol–gel combustion derived CaAl_2_O_4_:Dy^3+^ (2.0 mol%). The emission wavelength is fixed at 574 nm for the PL excitation measurement. As can be seen in Fig. 5, there are 6 peaks in the PL excitation spectrum of CaAl_2_O_4_:Dy^3+^ (2.0 mol%), which are located at 296, 326, 350, 389, 425, and 452 nm, respectively. The excitation peaks can be assigned to the 4f–4f transitions of Dy^3+^.^23–25^ The strongest absorption of CaAl_2_O_4_:Dy^3+^ is located at 350 nm, which is due to the ^6^H_15/2_ → ^6^P_7/2_ transition of Dy^3+^ activator. When compared to the strongest absorption at 350 nm, CaAl_2_O_4_:Dy^3+^ exhibits weaker absorptions at 296, 326, 389, 425 and 452 nm. These absorptions are due to ^6^H_15/2_ → ^4^D_7/2_, ^6^H_15/2_ → ^6^P_3/2_, ^6^H_15/2_ → ^4^I_13/2_, ^6^H_15/2_ → ^4^G_11/2_ and ^6^H_15/2_ → ^4^I_15/2_ transitions of Dy^3+^, respectively.^26–31^ The PL excitation spectrum indicates that a light source with the emission wavelength of 350 nm excites CaAl_2_O_4_:Dy^3+^ most efficiently. As a contrast, a light source with the emission wavelength of 326 nm can excite CaAl_2_O_4_:Dy^3+^ but in a much less efficient manner.

### 3.5 PL spectra of CaAl_2_O_4_:Dy^3+^ at different doping concentrations

**Fig. 6:**
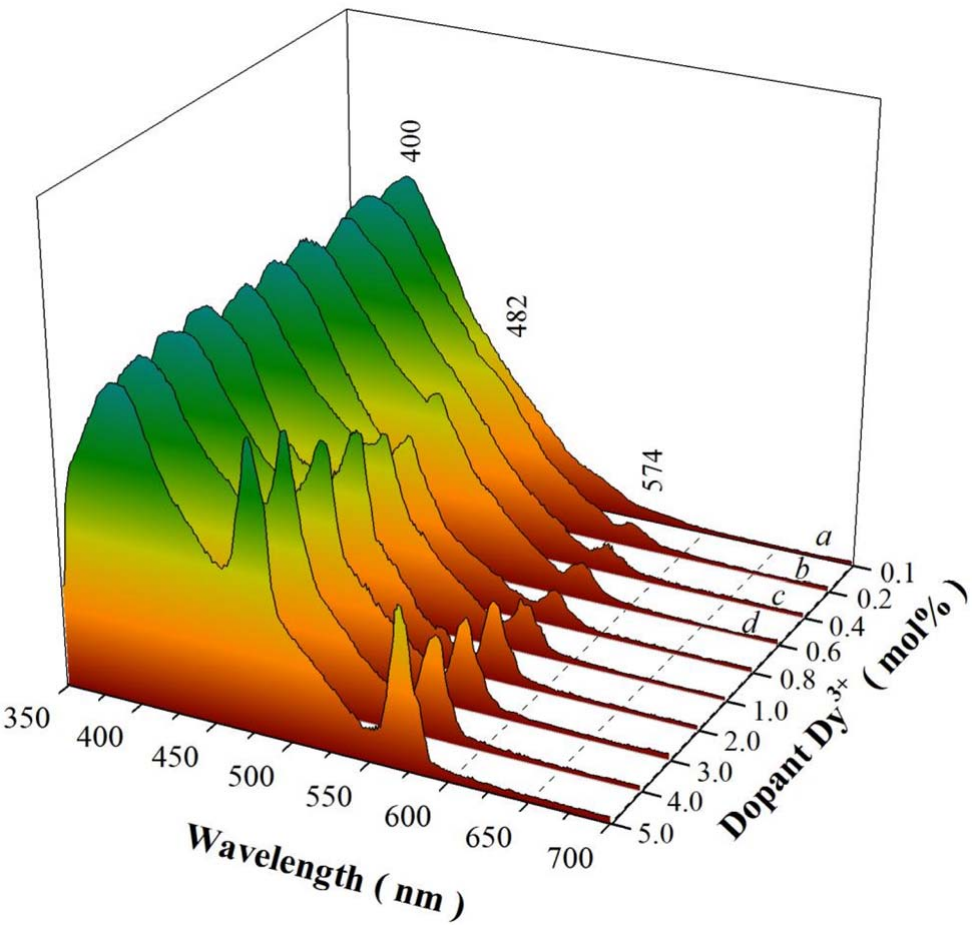
PL spectra of sol–gel combustion derived CaAl_2_O_4_:Dy^3+^ with doping concentrations in the range of 0.1–5.0 mol%. Excitation wavelength: 325 nm.

Fig. 6 represents the PL spectra of sol–gel combustion derived CaAl_2_O_4_:Dy^3+^ with doping concentrations in the range of 0.1–5.0 mol%. When the doping concentration is as low as 0.1 mol%, the PL spectrum of CaAl_2_O_4_:Dy^3+^ consists of a broad PL band peaking at about 400 nm whereas the characteristic emissions of Dy^3+^ are hardly discernible, as shown by the PL spectrum (a). The broadband emissions can be attributed to the intrinsic defect emissions in CaAl_2_O_4_, which are namely the oxygen and calcium vacancies.^3,4^ The PL properties of undoped CaAl_2_O_4_ and their origins were discussed in details in our previous work.^15,16^ Actually, similar broadband emissions are recorded for undoped CaAl_2_O_4_,^15,16^ Tb^3+^ doped CaAl_2_O_4_,^3,4^ undoped SrAl_2_O_4_,^8^ and Dy^3+^ doped BaAl_2_O_4_.^20^ The characteristic emissions of Dy^3+^ activator are represented by two narrowband emissions peaking at around 482 and 574 nm, respectively, which are due to the ^4^F_9/2_ → ^6^H_15/2_ and the ^4^F_9/2_ → ^6^H_13/2_ transitions of Dy^3+^ activator.^2–4,6,23^ As documented in the literature, the transition ^4^F_9/2_ → ^6^H_15/2_ (Δ*L* = 2 and Δ*J* = 3) is magnetic dipole allowed whereas the transition ^4^F_9/2_ → ^6^H_13/2_ is identified as a hypersensitive electric dipole transition (Δ*L* = 2 and Δ*J* = 2) of Dy^3+^. The reason why the characteristic emissions of Dy^3+^ activator are hardly discernible in the PL spectrum of CaAl_2_O_4_:Dy^3+^ (0.1 mol%) rests on the fact that the characteristic emissions of Dy^3+^ are too weak when compared to the strong emissions from the intrinsic defects in the host. As the doping concentration is increased to 0.2 and 0.4 mol%, the narrowband emissions of Dy^3+^ at 574 nm become discernible in the PL spectra of CaAl_2_O_4_:Dy^3+^ (0.2 and 0.4 mol%), as shown by the PL spectra (b) and (c). As the doping concentration is elevated further to 0.6 mol%, the narrowband emissions of Dy^3+^ can be identified clearly in the PL spectrum of CaAl_2_O_4_:Dy^3+^, as shown by the PL spectrum (d). It is apparent in Fig. 6 that the intensity of the characteristic emissions of Dy^3+^ increases monotonically with the doping concentration. Consequently, the PL spectrum of CaAl_2_O_4_:Dy^3+^ indicates that the intrinsic defects in CaAl_2_O_4_ and the doping species Dy^3+^ act as two independent sets of luminescence center of PL in CaAl_2_O_4_:Dy^3+^. The insight reason for the change in the PL intensity with doping of Dy^3+^ is due to the changes in the population density of the luminescence center of PL. The substitution of Dy^3+^ for Ca^2+^ in the lattice of CaAl_2_O_4_ promotes the production of two kinds of luminescence center of PL, as shown in eqn (1). The first kind of luminescence center of PL is the Dy^3+^ in Ca^2+^ site while the second kind of luminescence center of PL is the oxygen vacancy. As the doping concentration of Dy^3+^ increases, the population densities of the two kinds of luminescence center of PL are increased, which in turn lead to the enhancement in the PL intensities. It is noted that the PL intensity of each spectrum in Fig. 6 is normalized at 400 nm.1
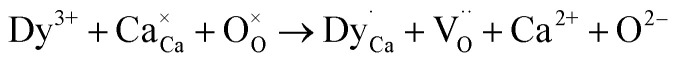


To check the influences of excitation wavelength on the emissions of CaAl_2_O4:Dy^3+^, we measured the PL spectra of CaAl_2_O_4_:Dy^3+^ (5 mol%) under different excitation wavelengths. The emission spectra of CaAl_2_O_4_:Dy^3+^ under the excitations of 326 nm and 350 nm are shown in Fig. S3.† As can be seen in Fig. S3,† the structures of the two PL spectra are nearly identical to each other regardless the variation in the excitation wavelength. However, the emission intensity of CaAl_2_O_4_:Dy^3+^ (5 mol%) is apparently sensitive to the excitation wavelength. For example, the characteristic emissions of Dy^3+^ are stronger under the excitation of 350 nm than those under the excitation of 326 nm. One of the reasons rests on the fact that Dy^3+^ exhibits stronger absorption at 350 nm than at 326 nm, as endorsed by Fig. 5.

Due to the combined contributions of defect emissions from the host and the characteristic emissions of the dopant, the color coordinates of the emissions of CaAl_2_O_4_:Dy^3+^ varies with the doping concentration. Color coordinates of luminescent materials, which can be calculated from their PL spectral data, are important parameters to quantitatively describe the emission color for luminescent materials.^32,33^ The CIE chromaticity coordinates of CaAl_2_O_4_:Dy^3+^ are given in Table S1† for different doping concentrations. As shown in Table S1,† the chromaticity coordinates of CaAl_2_O_4_:Dy^3+^ change with the doping concentration. For example, the chromaticity coordinates of CaAl_2_O_4_:Dy^3+^ (0.1 mol%) are (0.156, 0.094), those of CaAl_2_O_4_:Dy^3+^ (5.0 mol%) are (0.187, 0.176). The emission photos and the CIE chromaticity diagram of CaAl_2_O_4_:Dy^3+^ are shown in Fig. S4.† It is clear that the PL color of CaAl_2_O_4_:Dy^3+^ keeps blue when the doping concentration varies in the range of 0.1–5.0 mol%. As referred to the PL spectrum in Fig. 6, the defect-related emissions (blue) of the host are a major constituent in the PL spectrum whereas the characteristic emissions of Dy^3+^ are a minor constituent. That is the reason why the sol–gel combustion derived CaAl_2_O_4_:Dy^3+^ exhibits blue colored emission in spite of the variation of the doping concentration in the range of 0.1–5.0 mol%.

**Fig. 7:**
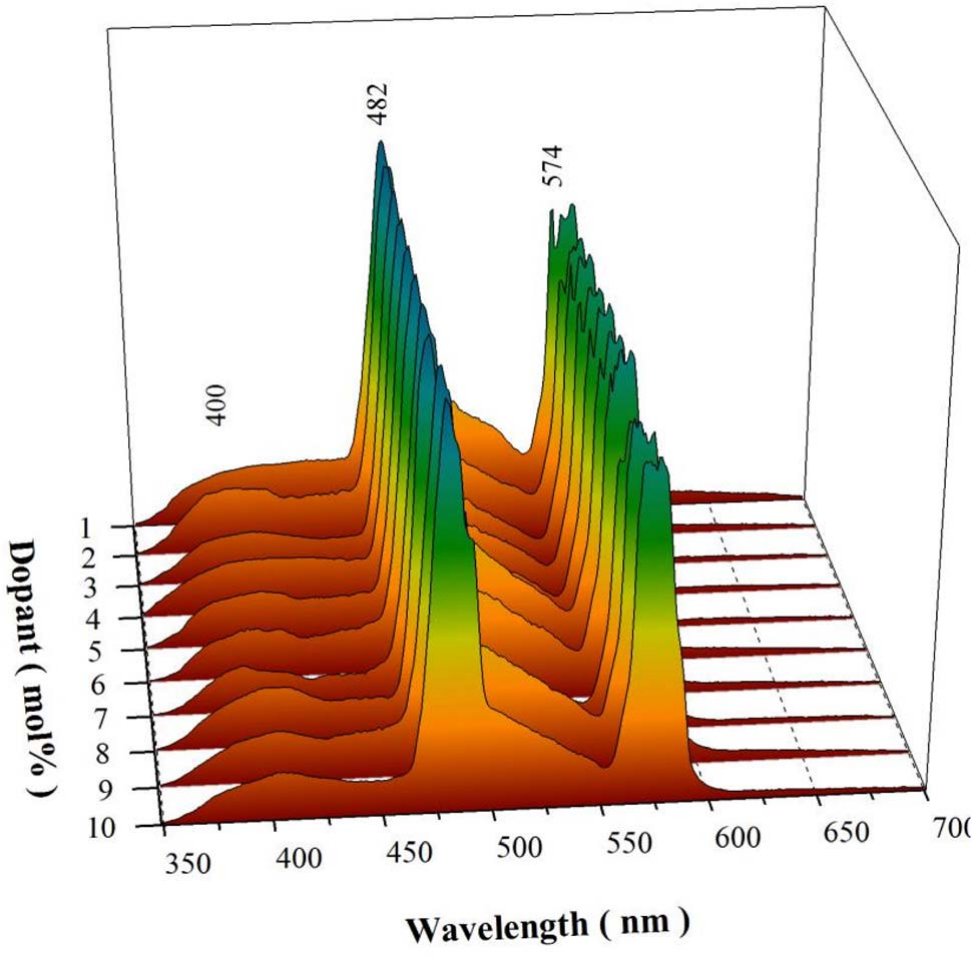
Normalized PL spectra of solid state reaction derived CaAl_2_O_4_:Dy^3+^ at 1350 °C for 10 h.

The assignment of the broadband emissions in Fig. 6 to intrinsic defects in CaAl_2_O_4_ gains further support from the PL spectrum of solid state reaction derived CaAl_2_O_4_:Dy^3+^. The solid state reaction route involves chemical decomposition and reactions at much high temperatures (often from 1000 to 1500 °C) to produce a new solid composition. At such high temperatures, oxygen atoms migrate into the crystal lattice to repair the defects with the result of decreased population density of oxygen vacancies in CaAl_2_O_4_. Therefore, the broadband emissions should be weakened if the density of oxygen vacancies in CaAl_2_O_4_ is reduced at high temperature. Fig. 7 depicts the normalized PL spectra of solid state reaction derived CaAl_2_O_4_:Dy^3+^ at 1350 °C for 10 h. Obviously, each PL spectrum of the solid state reaction derived CaAl_2_O_4_:Dy^3+^ consists of the characteristic emissions of Dy^3+^. The most striking feature in Fig. 7 is that the broadband emissions extending from 350 to 450 nm are weakened for the solid state reaction derived CaAl_2_O_4_:Dy^3+^. The chromaticity coordinates of the solid state reaction derived CaAl_2_O_4_:Dy^3+^ (10 mol%) are (0.270, 0.365), as shown in Fig. S5.†

### 3.6 Afterglow spectrum of CaAl_2_O_4_:Dy^3+^

**Fig. 8:**
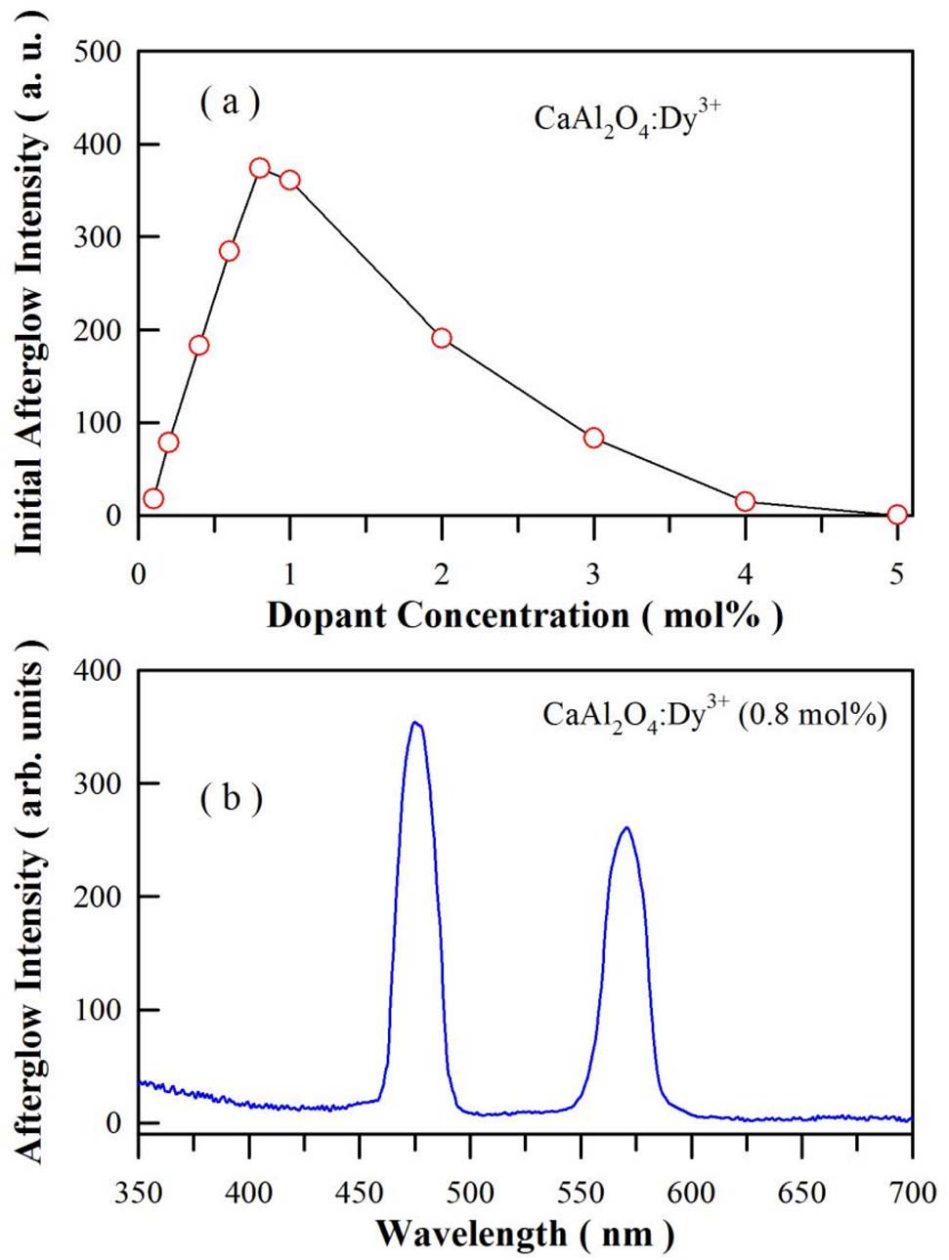
(a) Plot of the integrated afterglow intensity of sol–gel combustion derived CaAl_2_O_4_:Dy^3+^*versus* the dopant concentration of Dy^3+^; (b) afterglow spectrum of sol–gel combustion derived CaAl_2_O_4_:Dy^3+^ (0.8 mol%) at the doping concentration of 0.8 mol%. Before the afterglow measurement, the phosphor was exposed to the illumination of a high-pressure mercury lamp irradiation (175 W) for 3 min.

The emission spectrum of a high-pressure mercury lamp contains a pronounced spectral line at 365.4 nm, which is close to an absorption peak of CaAl_2_O_4_:Dy^3+^. After exposure to the illumination of a high-pressure mercury lamp (175 W) for 3 min, CaAl_2_O_4_:Dy^3+^ exhibits intense afterglow after the ultraviolet excitation is blocked off. Fig. S6† depicts the afterglow photos of CaAl_2_O_4_:Dy^3+^ (0.8 mol%) taken at different times after the irradiation of the high-pressure mercury lamp is blocked off. As shown in Fig. S6,† the white afterglow of CaAl_2_O_4_:Dy^3+^ (0.8 mol%) can last more than 60 min. The initial luminance of the afterglow is found to depend on the doping concentration of Dy^3+^. Fig. 8(a) shows the plot of the integrated afterglow intensity of sol–gel combustion derived CaAl_2_O_4_:Dy^3+^*versus* the doping concentration of Dy^3+^. As can be seen in Fig. 8(a), the optimal doping concentration is around 0.8 mol%. It is noted that the afterglow gets quenched when the doping concentration is high (*i.e.*, 5 mol%). Just like the case of PL quenching at high doping concentration, the non-radiative interaction between dopants is one of the reason of the concentration induced afterglow quenching.^34^ However, the traps generated by the dopant in the lattice of CaAl_2_O_4_ should be the key factor to be responsible for the afterglow quenching. As described in eqn (1), doping CaAl_2_O_4_ with Dy^3+^ results in oxygen vacancies. These positively charged oxygen vacancies can act as electron traps. The increase in the population density as well as the change in the trap depth of these the positively charged oxygen vacancies generate significant effects on the afterglow duration of the phosphor. For example, the afterglow duration is very short when the trap depth is shallow (*E* < 0.6 eV),^8,15,16,35^ and no afterglow can be observed when the trap depth is too deep (*E* > 2.0 eV). To observe afterglow at room temperature, the traps should have an appropriate activation energy somewhere between these two extremes, a trap depth around 0.65 eV is considered to be optimal.^7^Fig. 8(b) depicts the afterglow spectrum of the sol–gel combustion derived CaAl_2_O_4_:Dy^3+^ at the doping concentration of 0.8 mol%. As can be seen in Fig. 8(b), the afterglow spectrum consists of two narrow emission bands of Dy^3+^, which is distinctly different from the broadband afterglow of CaAl_2_O_4_:Eu^2+^.^1,21,36,37^ Thus Fig. 8(b) demonstrates that Dy^3+^ acts as the luminescence center of afterglow for CaAl_2_O_4_:Dy^3+^. The CIE chromaticity coordinates of the afterglow of CaAl_2_O_4_:Dy^3+^ (0.8 mol%) are calculated to be (0.265, 0.305), as shown in Fig. S7.† It is clear that the afterglow color of CaAl_2_O_4_:Dy^3+^ is close to white at the doping level 0.8 mol%. For comparison, the afterglow spectrum of solid state reaction derived CaAl_2_O_4_:Dy^3+^ (0.8 mol%) is given in the ESI as Fig. S8.†

### 3.7 Afterglow decay profile of CaAl_2_O_4_:Dy^3+^

**Fig. 9:**
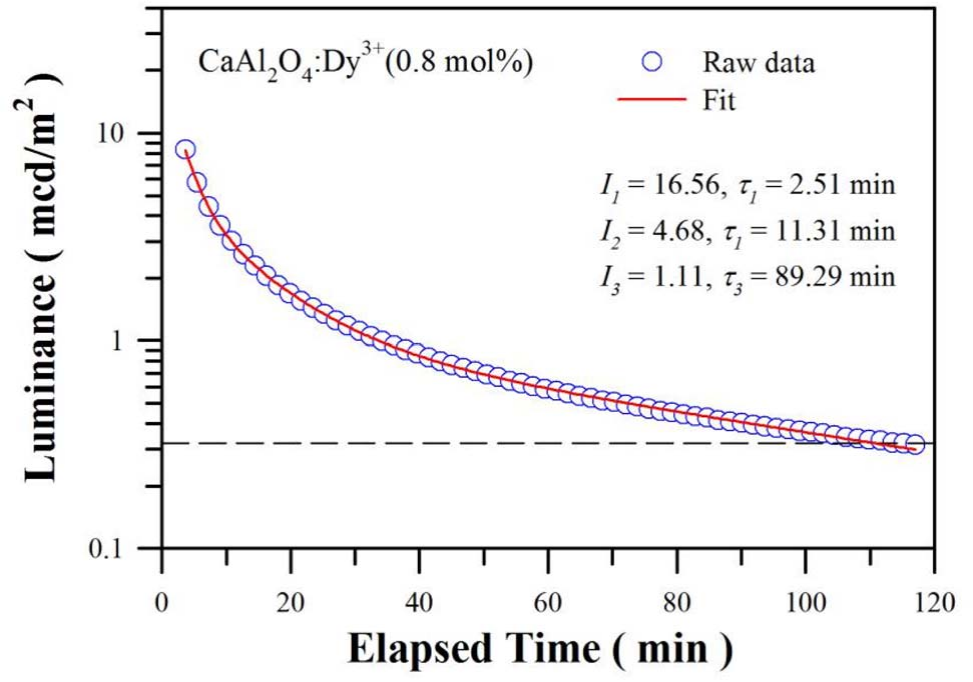
Afterglow decay profile of sol–gel combustion derived CaAl_2_O_4_:Dy^3+^ (0.8 mol%). The dash line represents the threshold luminance of 0.32 mcd m^−2^.

The duration of afterglow is a straightforward and standardized parameter to evaluate the properties of an afterglow material, whereby 0.32 mcd m^−2^ is often used as threshold for defining the duration of an afterglow. Fig. 9 depicts the afterglow decay profile of sol–gel combustion derived CaAl_2_O_4_:Dy^3+^ (0.8 mol%). The phosphor was exposed to illumination of a high-pressure mercury lamp irradiation (175 W) for 3 min before the measurement of afterglow decay curve. As shown by the raw data in Fig. 9, the afterglow duration of CaAl_2_O_4_:Dy^3+^ is determined to be about 115 min. It is found that the decay curves in Fig. 9 could be best fitted to the tri-exponential function according to eqn (2)

2 *I*(*t*) = *I*_1_ exp(−*t*/*τ*_1_) + *I*_2_ exp(−*t*/*τ*_2_) + *I*_3_ exp(−*t*/*τ*_3_),

where *I*(*t*) is the afterglow intensity at time *t* after blocking the laser excitation, *I*_*i*_ is the prefactor of the exponential component whose lifetime decay constant is *τ*_*i*_ (*i* = 1–3). The red solid line in Fig. 9 represents the fit of the experimental signals by eqn (2). The fitting parameters are tabulated in the figure. Clearly, the CaAl_2_O_4_:Dy^3+^ has three largely different decay components with constants of *τ*_1_ = 2.51 min, *τ*_2_ = 11.31 min and *τ*_3_ = 89.29 min. We can see that *τ*_2_ and *τ*_3_ are much longer than *τ*_1_, suggesting the presence of deeper traps in the phosphor. The luminance reading of the first data point in Fig. 9 is 8.3 mcd m^−2^ at 3.67 min. In order to derive the luminance at *t* = 0, it is necessary to extrapolate the data, yielding the value of the luminance to be 22.35 mcd m^−2^ at *t* = 0. It is worth of noting that the luminance of the phosphor at the initial moment is different from the luminance of the first component at the initial moment (*i.e.*, *I*_1_ = 16.56 mcd m^−2^). Actually, the luminance of the phosphor at the initial moment is the summation of the luminance of the three components at the initial moment, that is, the sum of *I*_1_, *I*_2_ and *I*_3_. Moreover, the afterglow duration of the phosphor is different from the longest lifetime decay constant *τ*_3_ (*i.e.*, 89.29 min). According to eqn (1), the afterglow duration of the phosphor is defined by the variable *t* when *I*(*t*) reaches the threshold 0.32 mcd m^−2^. The luminance decreases to 0.3205 mcd m^−2^ when *t* = 111 min. Consequently, the afterglow duration derived from eqn (2) (about 111 min) is very close the afterglow duration derived from raw data (about 115 min).

Being distinctly different from PL decay, the afterglow phenomenon is a particular case of thermostimulated luminescence and is a defect dependent phenomenon.^7,38^ Additional notes can be found in the ESI.† The afterglow duration of CaAl_2_O_4_:Dy^3+^ heavily depends on the wavelength of ultraviolet excitation. The afterglow of CaAl_2_O_4_:Dy^3+^ (0.8 mol%) lasts for about 60 s when the 325 nm excitation of the He–Cd laser (13 mW) is switched off. Conversely, the afterglow of CaAl_2_O_4_:Dy^3+^ (0.8 mol%) lasts for only 30 s when the 254 nm irradiation of a low-pressure mercury lamp (32 W) is turned off. Moreover, the afterglow duration of CaAl_2_O_4_:Dy^3+^ depends on the doping concentration when the wavelength of ultraviolet excitation is fixed. For example, after exposed to the 254 nm irradiation of a low-pressure mercury lamp (32 W) for 5 min, the afterglows of CaAl_2_O_4_:Dy^3+^ last for about 5, 8, 14, 19, 30, 60, 120, 75, 10 and 0 s when the doping concentrations are 0.1, 0.2, 0.4, 0.6, 0.8, 1.0, 2.0, 3.0, 4.0 and 5.0 mol%, respectively.

### 3.8 Possible PL and afterglow mechanisms of CaAl_2_O_4_:Dy^3+^

CaAl_2_O_4_ is an insulator with a bandgap of around 6.7 eV.^39,40^ Oxygen and calcium vacancies are intrinsic defects in CaAl_2_O_4_:Dy^3+^. On one hand, these intrinsic vacancies act as luminescence center of PL with the result of a broadband PL spectrum peaking at about 400 nm.^15,16^ On the other hand, these intrinsic vacancies work as traps for charged carriers. For example, oxygen vacancies in CaAl_2_O_4_ are proposed to work as electron traps because they are positively charged whereas calcium vacancies are potential hole traps because they are positively charged.^10,39^ Dopant Dy^3+^ belongs to extrinsic defect in CaAl_2_O_4_:Dy^3+^. After Dy^3+^ ions are incorporated into the host, a series of defect energy levels are introduced into the bandgap of CaAl_2_O_4_. The lowest energy level of the excited state of Dy^3+^ is known as ^4^F_9/2_ while the energy levels of the ground state of Dy^3+^ are denoted as ^6^H_*J*_ (*J* = 15/2–5/2). As evidenced by the characteristic emissions in Fig. 6, 7 and S3,† dopant Dy^3+^ ions act as luminescent center of PL in CaAl_2_O_4_:Dy^3+^ to yield narrowband emissions peaking at 482 and 574 nm, respectively. Furthermore, this extrinsic defect can work as electron trap because it is positively charged.

**Fig. 10:**
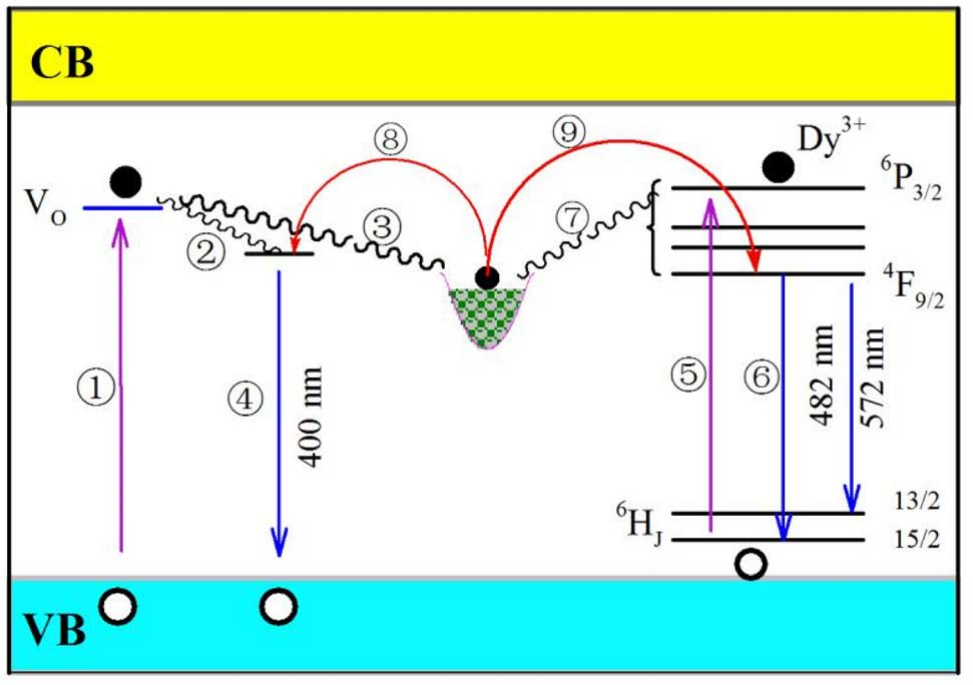
Schematic illustration on the PL and afterglow mechanisms of CaAl_2_O_4_:Dy^3+^. Process ①: host absorption of incident photons due to the presence of intrinsic defects in CaAl_2_O_4_. Process ②: non-radiative relaxation of hot electrons to oxygen vacancies (luminescence center) in CaAl_2_O_4_. Process ③: non-radiative relaxation of hot electrons to electron traps in CaAl_2_O_4_. Process ④: radiative recombination of electrons captured at oxygen vacancy with holes in the valence band to yield the broadband emissions peaking at about 400 nm. Process ⑤: absorption of incident photons due to the transition ^6^H_15/2_ → ^6^P_3/2_ of Dy^3+^ (around 325 nm) in CaAl_2_O_4_. Process ⑥: electron transitions ^4^F_9/2_ → ^6^H_15/2_ and ^4^F_9/2_ → ^6^H_13/2_ of Dy^3+^ to produce the characteristic emissions of Dy^3+^. Process ⑦: non-radiative relaxation of hot electrons at excited states of Dy^3+^ to electron traps in CaAl_2_O_4_. Process ⑧: thermal release of electrons from electron traps to oxygen vacancies in CaAl_2_O_4_. Process ⑨: thermal release of electrons from electron traps to the excited states of Dy^3+^.

Fig. 10 schematically illustrates the PL and afterglow mechanisms of CaAl_2_O_4_:Dy^3+^. The energy levels of oxygen vacancies and dopant Dy^3+^ are depicted in the bandgap of CaAl_2_O_4_. As shown in Fig. 10, oxygen vacancies and Dy^3+^ activator are two independent sets of photon absorbents in CaAl_2_O_4_:Dy^3+^. Upon the ultraviolet excitation at 325 nm (3.82 eV), a portion of the incident photons are absorbed by the host due to the presence of a large number of oxygen vacancies in CaAl_2_O_4_ (process ①). After non-radiative relaxations, the hot electrons are captured by either the oxygen vacancies (process ②) or the electron traps (process ③). The subsequent radiative recombination of electrons captured at oxygen vacancies with holes in the valence band of CaAl_2_O_4_ yields the broadband PL peaking at about 400 nm (process ④). Since the transition ^6^H_15/2_ → ^6^P_3/2_ of Dy^3+^ (326 nm) matches well with the 325 nm excitation of the laser, some incident photons are absorbed by Dy^3+^ activator in CaAl_2_O_4_ (process ⑤). It is noted that several energy levels are located between the excited state ^6^P_3/2_ and the lowest excited state ^6^H_15/2_, among which include ^4^I_13/2_, ^4^P_5/2_ and ^6^P_7/2_. With the assistance of phonons, a portion of hot electrons at the excited state ^6^P_3/2_ are relaxed to the lowest excited state ^4^F_9/2_. Then electron transitions ^4^F_9/2_ → ^6^H_15/2_ and ^4^F_9/2_ → ^6^H_13/2_ of Dy^3+^ lead to the characteristic emissions of Dy^3+^ (process ⑥). Apart from the radiation recombination, some hot electrons at excited states of Dy^3+^ can be captured by the electron traps *via* non-radiative relaxations (process ⑦). In the light of the proposed mechanisms in Fig. 10, the PL features of CaAl_2_O_4_:Dy^3+^ can be interpreted: (i) the PL spectrum of CaAl_2_O_4_:Dy^3+^ consists of one broadband and two characteristic emissions of Dy^3+^; and (ii) the characteristic emissions of Dy^3+^ becomes dominant over the broadband emissions when the population density of oxygen vacancies in CaAl_2_O_4_:Dy^3+^ is significantly suppressed *via* the solid state reaction at high temperatures.

Once the ultraviolet excitation is ceased, processes ①–⑦ are stopped immediately. Under thermal activation, electrons can be released from the electron traps *via* processes ⑧ and ⑨. Subsequently, afterglow with characteristic emissions of Dy^3+^ is resulted when the electrons released from the excited states of Dy^3+^ recombine radiatively with holes *via* process ⑥. Theoretically speaking, afterglow with broadband emissions peaking at about 400 nm should be observed when thermally detrapped electrons recombine radiatively with holes *via* process ④. In practice, such a broadband afterglow is negligible because its intensity is several orders of magnitude weaker than that of Dy^3+^ related afterglows. Therefore, the afterglow spectrum of the CaAl_2_O_4_:Dy^3+^ consists of two narrowband emissions peaking at 482 and 574 nm, respectively.

In the light of the PL and afterglow mechanisms of CaAl_2_O_4_:Dy^3+^ in Fig. 10, electrons in the electron traps contribute to the PL under photoexcitation, too. Upon the photoexcitation, some electrons in the electron traps are detrapped *via* processes ⑧ and ⑨. Broadband emissions peaking at around 400 nm can be expected when the detrapped electrons recombine radiatively with holes *via* the radiative process ④, and narrowband emissions of Dy^3+^ can be resulted when the detrapped electrons recombine radiatively with holes *via* the radiative process ⑥. In most cases, however, such contributions are negligible because they are many times weaker than the emissions resulted directly from the ultraviolet photoexcitation. Only when the afterglow is sufficiently strong, such contributions to the PL are discernible. For example, the PL spectrum of Dy^3+^ doped SrAl_2_O_4_ is the result of superposition of the broadband emissions of the host peaking at about 400 nm and another broadband emissions peaking at about 520 nm.^12,13^

### 3.9 TL glow curves of CaAl_2_O_4_:Dy^3+^

**Fig. 11:**
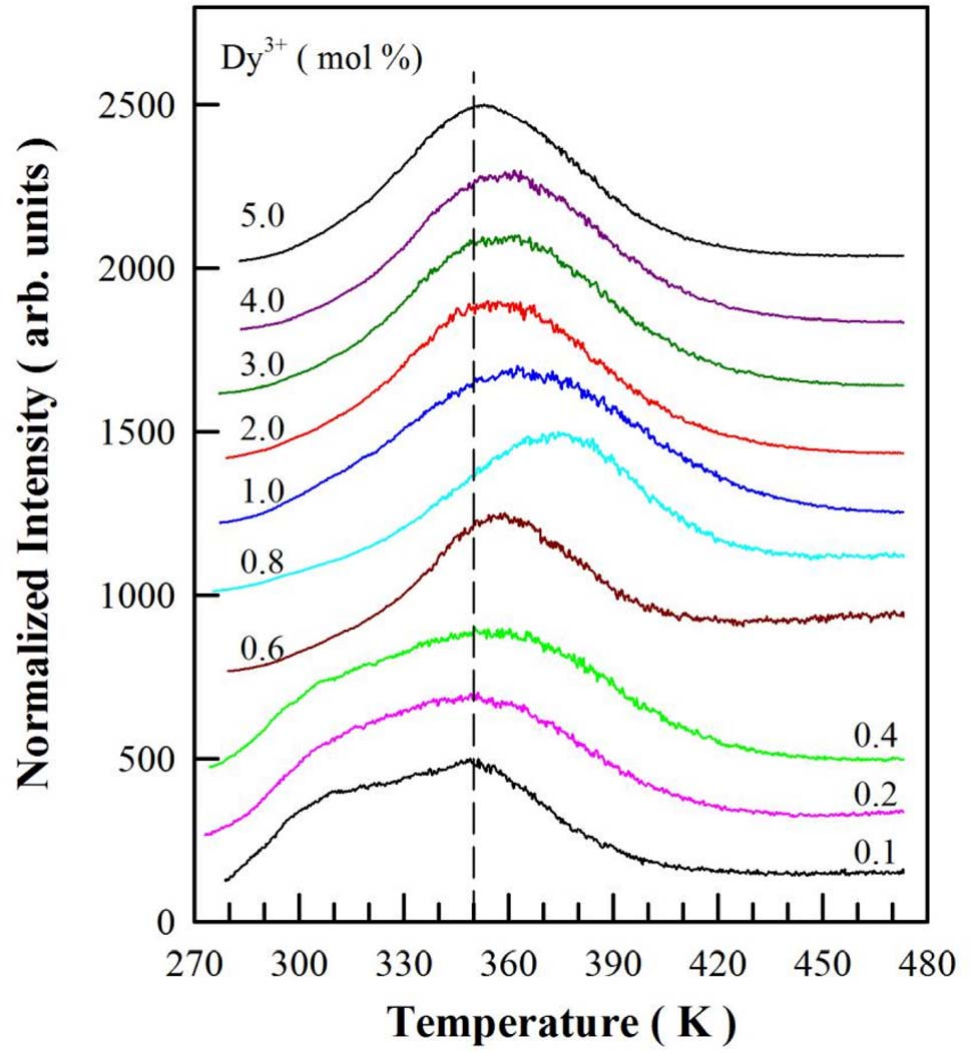
TL glow curves of sol–gel combustion derived CaAl_2_O_4_:Dy^3+^ with different doping concentrations. CaAl_2_O_4_:Dy^3+^ phosphors were exposed to ultraviolet light of 254 nm for 5 min before the TL measurement.

TL is an important tool to determine the activation energies (*i.e.*, trap depths) of trapping levels in crystals.^37,39,41,42^Fig. 11 represents the TL glow curves of sol–gel combustion derived CaAl_2_O_4_:Dy^3+^ with different doping concentrations. Each CaAl_2_O_4_:Dy^3+^ phosphor was exposed to ultraviolet light of 254 nm for 5 min before the TL measurements. The temperature rising rate was 2 K min^−1^. It can be seen that each TL glow curve in Fig. 11 exhibits an extremely broad and asymmetric profile, suggesting the presence of multiple trap levels in CaAl_2_O_4_:Dy^3+^.^43^ Moreover, both the profile and the peak temperature of the TL glow curve are sensitive to the doping concentration: (i) the TL glow curve consists of a primary peak at around 350 K and a secondary peak at about 310 K when the doping concentration increases from 0.1 to 0.4 mol%; (ii) the TL glow curve has only one peak, which gradually shifts to higher temperature (from 350 to 375 K) as the doping concentration increases from 0.6 to 0.8 mol%; and (iii) the TL glow curve has only one peak, which gradually shifts to lower temperature (from 375 to 350 K) as the doping concentration increases further from 1.0 to 5.0 mol%. The vertical dash line in Fig. 11 marks the position of 350 K. With the vertical line as a guideline, the evolution of the peak temperature with the doping concentration can be identified clearly. Interestingly, the peak temperature of the TL glow curve of CaAl_2_O_4_:Dy^3+^ (0.8 mol%) is the highest (around 375 K) among the 10 phosphors under test, which coincides with the best afterglow performance of CaAl_2_O_4_:Dy^3+^ at optimal doping concentration of 0.8 mol%.

**Fig. 12:**
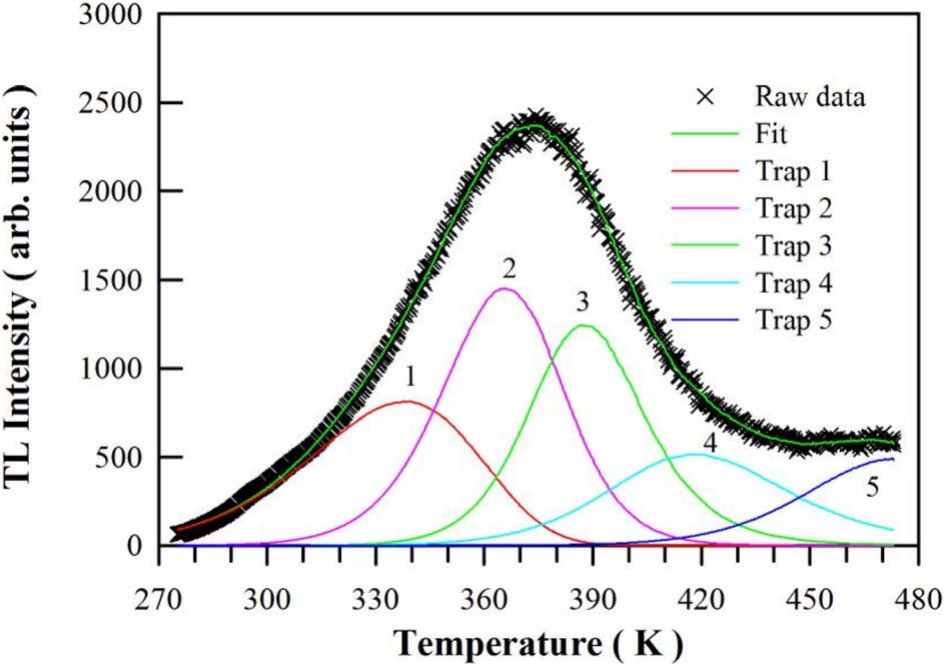
Computerized glow curve deconvolution of the TL glow curve of CaAl_2_O_4_:Dy^3+^ (0.8 mol%) under the assumption of 5 traps in the phosphor.

To understand the TL behavior of CaAl_2_O_4_:Dy^3+^, it is necessary to deconvolute each TL glow curve and evaluate the trapping parameters. The TL curve based on the general order function is given by the following equation:3
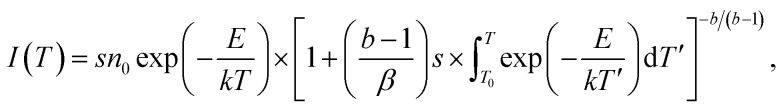
where *I* is the TL intensity at temperature *T*, *s* is the pre-exponential factor with the unit of s^−1^, *n*_0_ is the concentration of trapped charges at time *t* = 0, *E* is the trap depth, *k* is the Boltzmann constant, *b* is the order of kinetics, and *β* is the heating rate. In order to determine the kinetic parameters of the multiple traps, computerized glow curve deconvolution of the TL glow curves is carried out with general order kinetics using a computer program given by Chung *et al.*^44^Fig. 12 depicts the computerized glow curve deconvolution of the TL glow curve of CaAl_2_O_4_:Dy^3+^ at the doping concentration of 0.8 mol%. It is found that this TL glow curve can be described satisfactorily by using the general order kinetics to model 5 traps in CaAl_2_O_4_:Dy^3+^. The figure-of-merit (FOM) of the deconvolution is 2.071%. The kinetic parameters and the electron lifetime at room temperature (*τ*_300_) are summarized in Table 1 for each trap in the CaAl_2_O_4_:Dy^3+^ (0.8 mol%). As can be seen in Table 1, the calculated *E* values are 0.3919, 0.8219, 1.1466, 0.8403 and 1.0352 eV for the five traps in CaAl_2_O_4_:Dy^3+^ (0.8 mol%). Apparently, most of these traps are suitable for long-time afterglow.

**Table 1:** Kinetic parameters of the computerized glow curve deconvolution of the TL glow curve of CaAl_2_O_4_:Dy^3+^ (0.8 mol%). *T*_m_ represents the peak temperature, *E* is the trap-depth, *s* is the frequency factor, *b* is the order of kinetics, and *τ*_300_ is the room temperature electron lifetime in the trap

Trap number	*T*_m_ (K)	*E* (eV)	*s* (s^−1^)	*b*	*τ*_300_ (h)	FOM (%)
1	339.25	0.3919	9.234 × 10^2^	1.000	1.93 × 10^0^	2.071
2	365.55	0.8219	4.810 × 10^8^	1.482	2.11 × 10^2^	
3	386.45	1.1466	2.256 × 10^12^	2.000	1.02 × 10^6^	
4	418.95	0.8403	2.336 × 10^7^	2.000	4.71 × 10^5^	
5	472.85	1.0352	1.683 × 10^8^	2.000	1.59 × 10^8^	

In addition to the TL glow curve shown in Fig. 12, we have also deconvoluted the TL glow curves of CaAl_2_O_4_:Dy^3+^ with the doping concentrations of 0.1, 0.2, 0.4, 0.6, 1.0, 2.0, 3.0, 4.0 and 5.0 mol%, respectively. For the sake of brevity, the computerized glow curve deconvolutions of the TL glow curves of CaAl_2_O_4_:Dy^3+^ are shown in ESI as Fig. S9–S17.† The kinetic parameters and the electron lifetime at room temperature are listed in Table S2† for each trap in the CaAl_2_O_4_:Dy^3+^. Apparently, the parameters of electron traps in CaAl_2_O_4_:Dy^3+^ can be effectively tuned *via* the control of doping concentration, which in turn can be exploited to modify the brightness and duration of the afterglow of CaAl_2_O_4_:Dy^3+^.

## 4. Conclusions

With doping concentration varying in the range of 0.1–5.0 mol%, a series of CaAl_2_O_4_:Dy^3+^ phosphors have been synthesized *via* sol–gel combustion technique. The PL and afterglow mechanisms of sol–gel combustion derived CaAl_2_O_4_:Dy^3+^ are explored by means of XRD, SEM, TEM, EDX, XPS, PL, afterglow spectroscopy and TL dosimetry. The PL spectrum of CaAl_2_O_4_:Dy^3+^, which consists of a broad PL band peaking at about 400 nm and the characteristic emissions of Dy^3+^, indicates that oxygen vacancies and Dy^3+^ work as two independent sets of luminescence center of PL for CaAl_2_O_4_:Dy^3+^. As a contrast, the afterglow spectrum of CaAl_2_O_4_:Dy^3+^ consists of the characteristic emissions of Dy^3+^ only, verifying that dopant Dy^3+^ acts as the luminescence center of afterglow for CaAl_2_O_4_:Dy^3+^. The afterglow duration of CaAl_2_O_4_:Dy^3+^ is found to depend on the doping concentration, and 115 min-long afterglow is recorded for CaAl_2_O_4_:Dy^3+^ at the optimal doping concentration of 0.8 mol%. The profile and peak temperature of the TL glow curve are sensitive to the doping concentration. The TL glow curve of CaAl_2_O_4_:Dy^3+^ (0.8 mol%) exhibits a maximal peak temperature at around 375 K. A picture on the PL and afterglow mechanisms is given to reveal the processes of excitation, migration, trapping, detrapping, and radiative recombination of charged carriers in CaAl_2_O_4_:Dy^3+^.

## Conflicts of interest

There are no conflicts to declare.

## Supplementary Material

RA-012-D2RA05008K-s001
