# Supplementary material for: Photoluminescence and afterglow of Dy3+ doped CaAl2O4 derived via sol–gel combustion
Source: RSC Adv. 2022 Nov 8;12(49):31757–68. doi: 10.1039/d2ra05008k (PMC9641381; doi:10.1039/d2ra05008k)
Supplement: RA-012-D2RA05008K-s001 [file RA-012-D2RA05008K-s001.pdf]

Electronic Supplementary Material (ESI) for RSC Advances.
This journal is © The Royal Society of Chemistry 2022

Supplementary Material

Photoluminescence and afterglow of Dy^{3+} doped $CaAl_2O_4$ derived via sol-gel combustion

Bao–gai Zhai, Meng Meng Chen, Yuan Ming Huang*

School of Microelectronics and Control Engineering, Changzhou University, Changzhou 213164, China

Additional Notes: Differences between PL lifetime and afterglow duration.

Figure S1. (a) Raw data of the XRD pattern $CaAl_2O_4:Dy^{3+}$ with the doping concentration of 0.1 mol%; (b) the XRD pattern $CaAl_2O_4$ registered in the JCPDF card no. 23-1036; (c) Calculated XRD pattern of monoclinic $CaAl_2O_4$.

Figure S2. (a) Conventional unit cell of monoclinic $CaAl_2O_4$ (viewing along *x* axis); (b) Crystal structure of monoclinic $CaAl_2O_4$ showing the channels formed by AlO_4 tetrahedra (viewing along *y* axis).

Figure S3. PL spectra of sol-gel combustion derived $CaAl_2O_4:Dy^{3+}$ under the ultraviolet excitation of 326 nm (black curve) and 350 nm (red curve).

Figure S4. PL photo and CIE chromaticity diagram to show the emissions of $CaAl_2O_4:Dy^{3+}$ (0.1−5.0 mol%) derived via sol-gel combustion. Excitation wavelength of the He-Cd laser is 325 nm.

Figure S5. CIE chromaticity diagram of the PL of $CaAl_2O_4:Dy^{3+}$ (10 mol%) derived via solid phase reaction.

Figure S6. (a) Photograph of the sol-gel combustion derived $CaAl_2O_4:Dy^{3+}$ (0.8 mol%) under lab illumination. (b) Afterglow photographs of the phosphor taken at different times after the removal of the irradiation of a high-pressure mercury lamp (175 W).

Figure S7. CIE chromaticity diagram to show the afterglow of $CaAl_2O_4:Dy^{3+}$ (0.6 mol%) derived via sol-gel combustion.

Figure S8. Afterglow spectrum of solid state reaction derived $CaAl_2O_4:Dy^{3+}$ (0.8 mol%).

Figure S9-S17. Computerized glow curve deconvolution of the TL glow curves of $CaAl_2O_4:Dy^{3+}$ (x = 0.1, 0.2, 0.4, 0.6, 1.0, 2.0, 3.0, 4.0, 5.0 mol%);

Table S1. CIE chromaticity coordinates of $CaAl_2O_4:Dy^{3+}$ with different doping concentrations.

Table S2. Kinetic parameters of the computerized glow curve deconvolution of the TL glow curve of $CaAl_2O_4:Dy^{3+}$ (0.1–5.0 mol%). T_m represents the peak temperature, E is the trap-depth, s is the frequency factor, b is the order of kinetics, and τ_{300} is the room temperature electron lifetime in the trap.

Additional Notes: Differences between PL lifetime and afterglow duration

The afterglow duration of 115 min indicates that, after the extinction of the ultraviolet excitation, the sample emits light for the duration of 115 min with decayed intensity to 0.32 mcd/m^2. The time-resolved PL spectrum of $CaAl_2O_4$:Dy^{3+} (0.8 mol%) shows that lifetime of Dy^{3+} emission at 574 nm is in the order of millisecond. Such a large difference between the afterglow duration and the PL lifetime can be accounted by the fact the PL and the afterglow are two distinctly different processes. The PL decay measures the electron depopulation of excited states, and PL lifetime is the time a fluorophore spends in the excited state before emitting a photon and returning to the ground state. As discussed before, the PL lifetime equals to the inverse of summation of the radiative and non-radiative rates. Generally, PL lifetime varies from picoseconds to hundreds of nanoseconds depending on the fluorophore. The afterglow measure the carrier depopulation from traps, and the afterglow duration of a material is governed by the reaps in the material. In the case of the characteristic emissions of Dy^{3+} in the lattice of $CaAl_2O_4$, the long PL lifetime in the order of millisecond is caused by the spin-forbidden transitions (i.e., the *4f–4f* transition). As a contrast, the long afterglow duration is caused by the storage of energy in traps that are slowly promoted to the luminescence center of afterglow. In afterglow materials, the energy is stored by trapping charged carriers (i.e., electrons and holes) in traps, which are slowly released from the traps upon thermal stimulation. Thus, afterglow is a particular case of thermostimulated luminescence and is a defect dependent phenomenon. Detailed discussions can be found in relevant reviews.

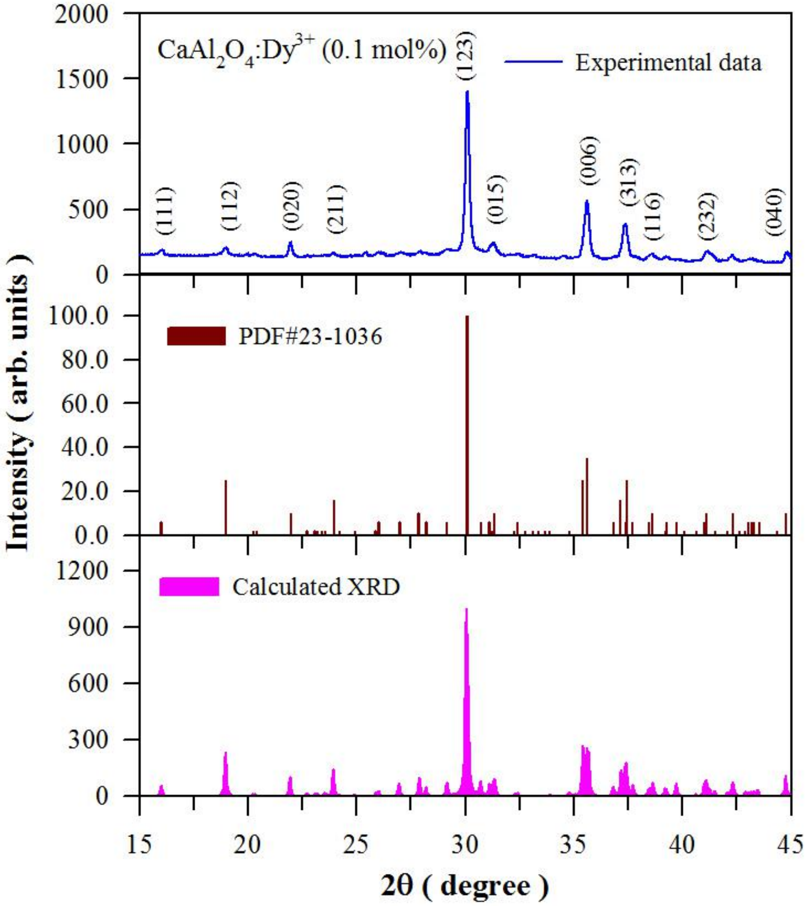

Figure S1. (a) Raw data of the XRD pattern $CaAl_2O_4:Dy^{3+}$ with the doping concentration of 0.1 mol%; (b) the XRD pattern $CaAl_2O_4$ registered in the JCPDF card no. 23-1036; (c) Calculated XRD pattern of monoclinic $CaAl_2O_4$.

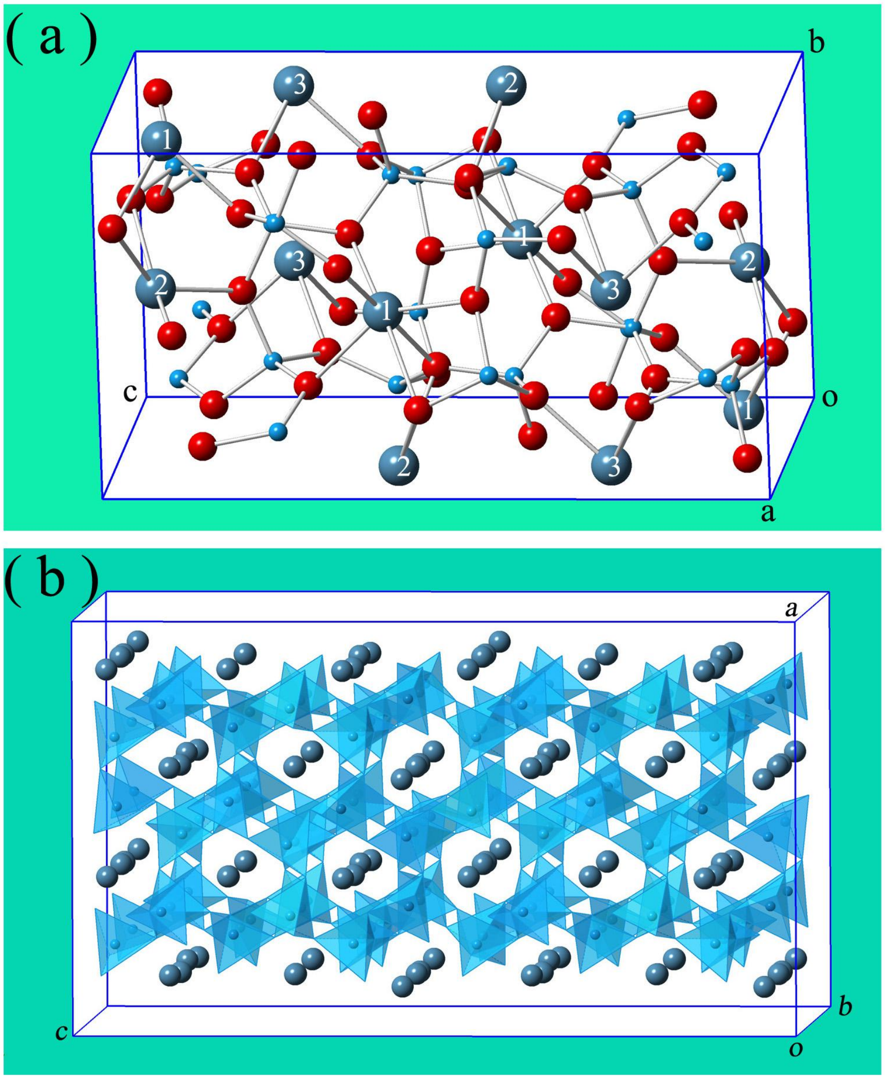

Figure S2. (a) Conventional unit cell of monoclinic $CaAl_2O_4$ (viewing along *x* axis); (b) Schematic view of the monoclinic $CaAl_2O_4$ along the *b*-direction to show the channels formed by AlO_4 tetrahedra.

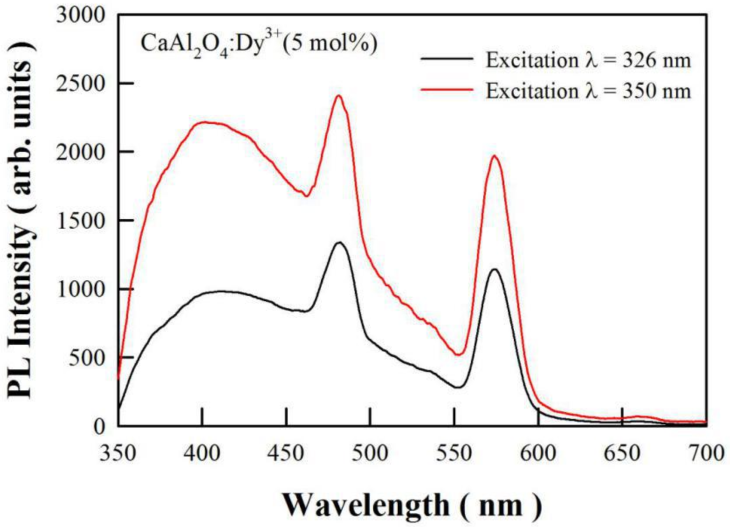

Figure S3. PL spectra of sol-gel combustion derived $CaAl_2O_4:Dy^{3+}$ under the ultraviolet excitation of 326 nm (black curve) and 350 nm (red curve).

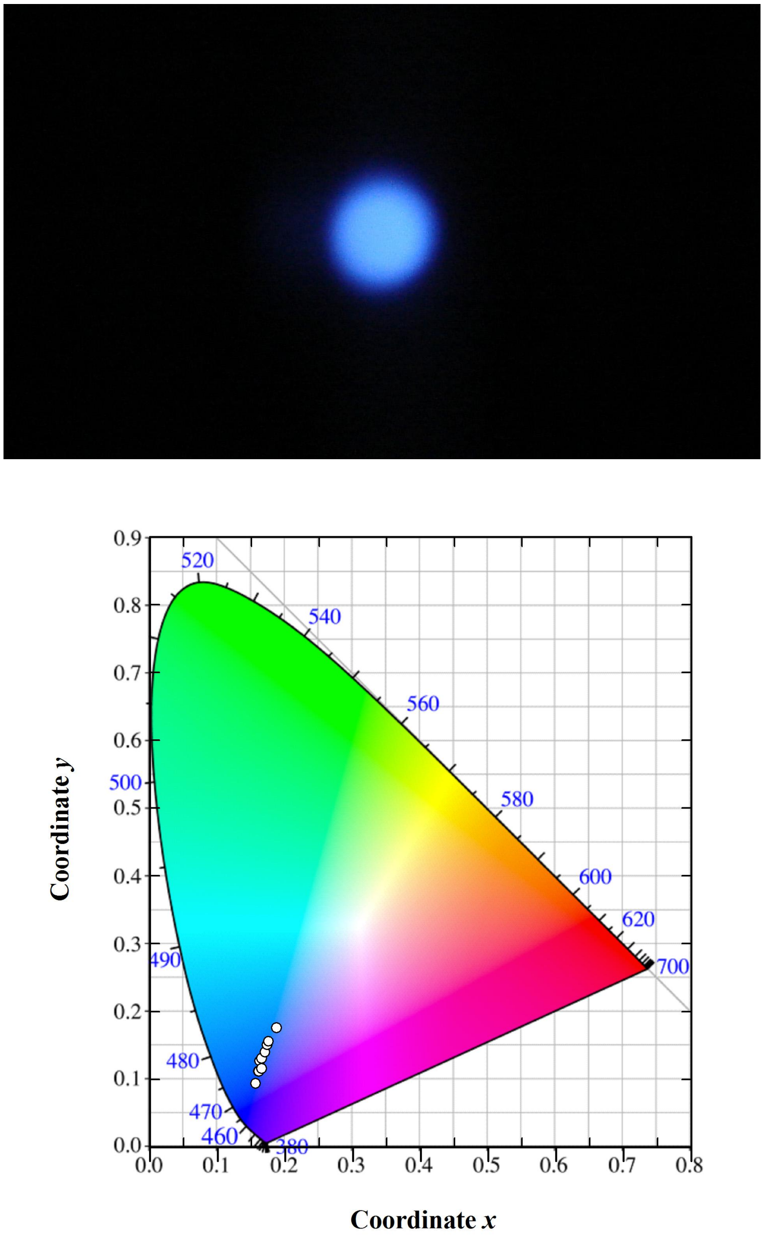

Figure S4. PL photo and CIE chromaticity diagram to show the emissions of $CaAl_2O_4:Dy^{3+}$ (0.1–5.0 mol%) derived via sol-gel combustion. Excitation wavelength of the He-Cd laser is 325 nm.

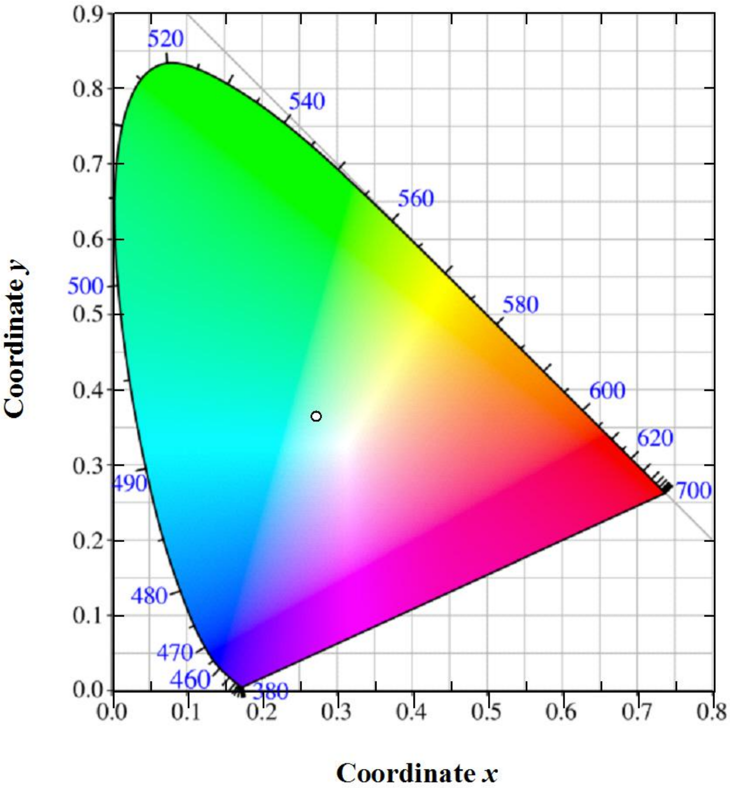

Figure S5. CIE chromaticity diagram of the PL of the solid state reaction derived $CaAl_2O_4:Dy^{3+}$ (10 mol%).

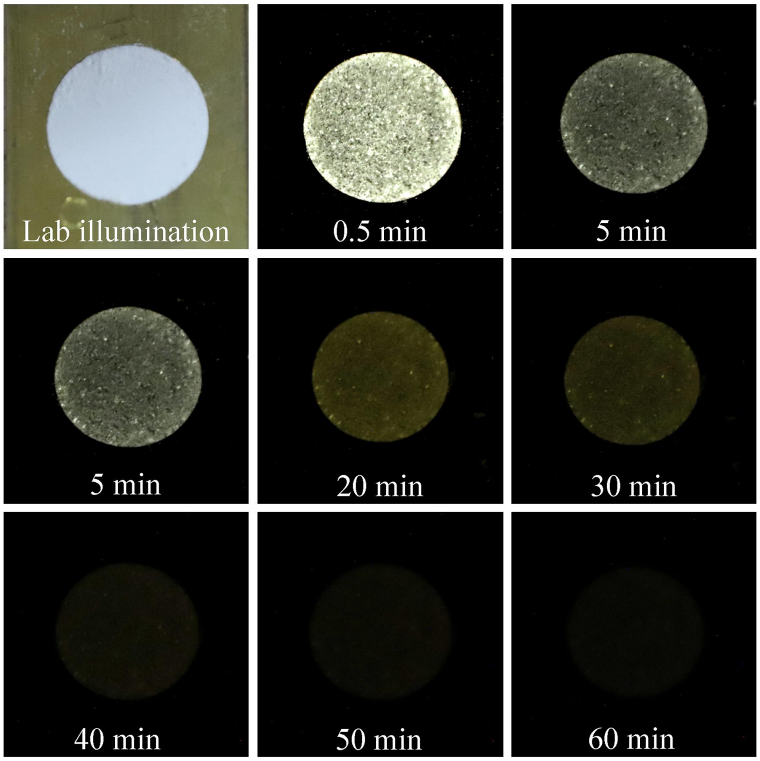

Figure S6. (a) Photograph of the sol-gel combustion derived $CaAl_2O_4:Dy^{3+}$ (0.8 mol%) under lab illumination. (b) Afterglow photographs of the phosphor taken at different times after the removal of the irradiation of a high-pressure mercury lamp (175 W). The phosphor was kept in a bronze container and exposed to the irradiation from a high-pressure mercury lamp (175 W) for 3 min before taking the afterglow photos. The diameter of each disc was 20 mm.

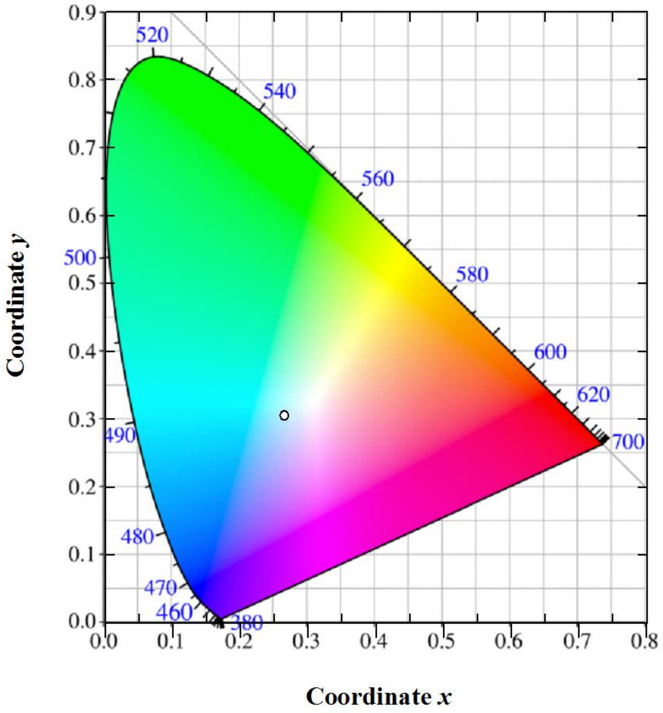

Figure S7. CIE chromaticity diagram to show the afterglow color of sol-gel combustion derived $CaAl_2O_4:Dy^{3+}$ (0.6 mol%).

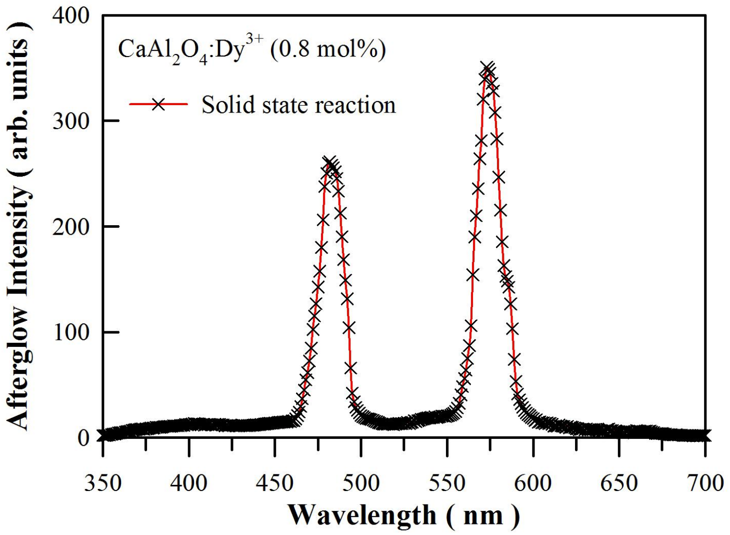

Figure S8. Afterglow spectrum of solid state reaction derived $CaAl_2O_4:Dy^{3+}$ (0.8 mol%). Before the afterglow measurement, the phosphor was exposed to the illumination of a high-pressure mercury lamp irradiation (175 W) for 3 min.

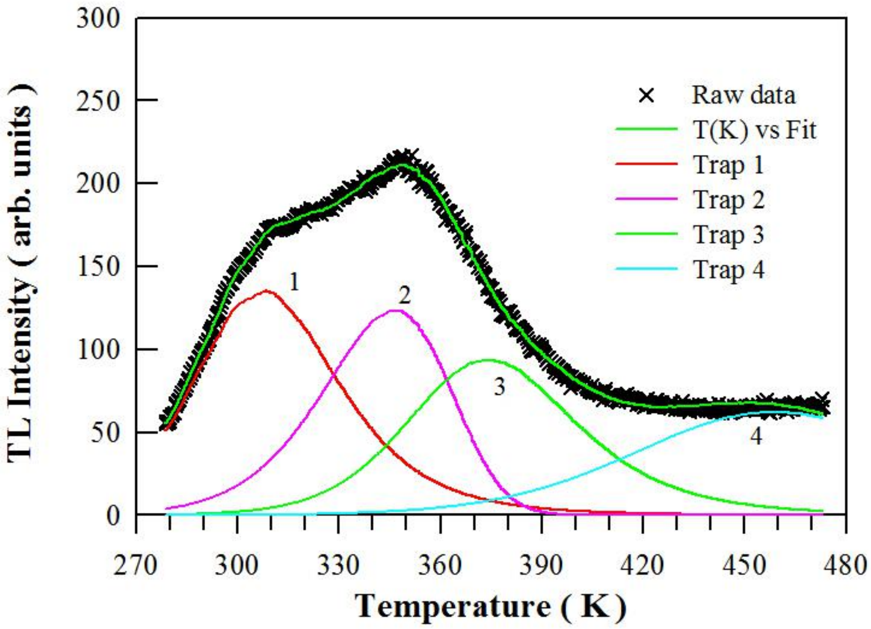

Figure S9. Computerized glow curve deconvolution of the TL glow curve of $CaAl_2O_4:Dy^{3+}$ (0.1 mol%) assuming the presence of 4 traps in the phosphor.

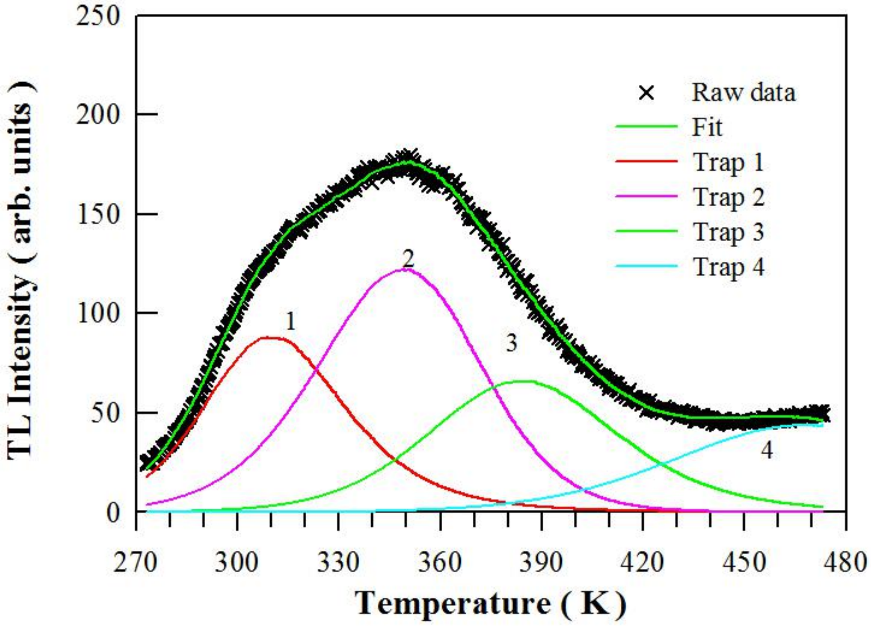

Figure S10. Computerized glow curve deconvolution of the TL glow curve of $CaAl_2O_4:Dy^{3+}$ (0.2 mol%) assuming the presence of 4 traps.

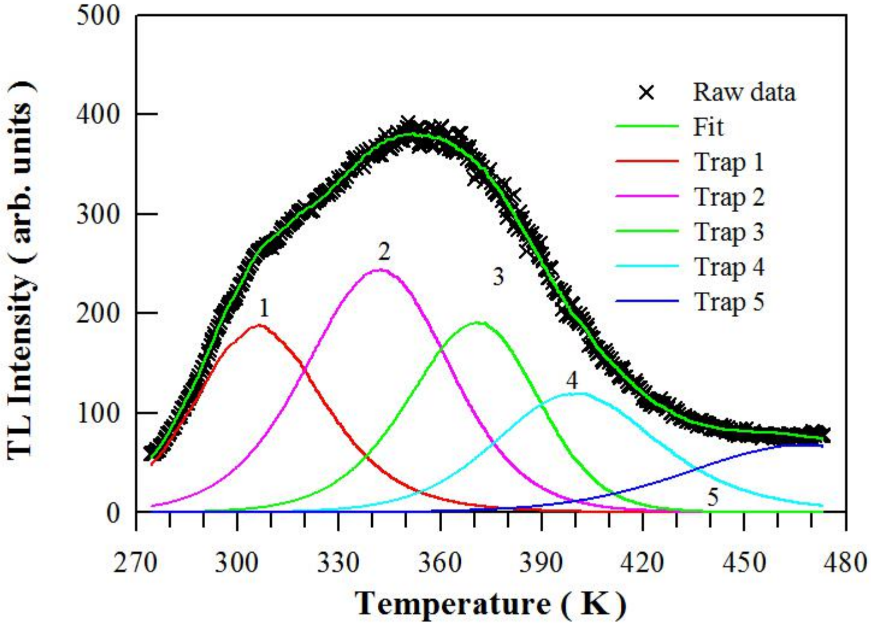

Figure S11. Computerized glow curve deconvolution of the TL glow curve of $CaAl_2O_4:Dy^{3+}$ (0.4 mol%) assuming the presence of 5 traps.

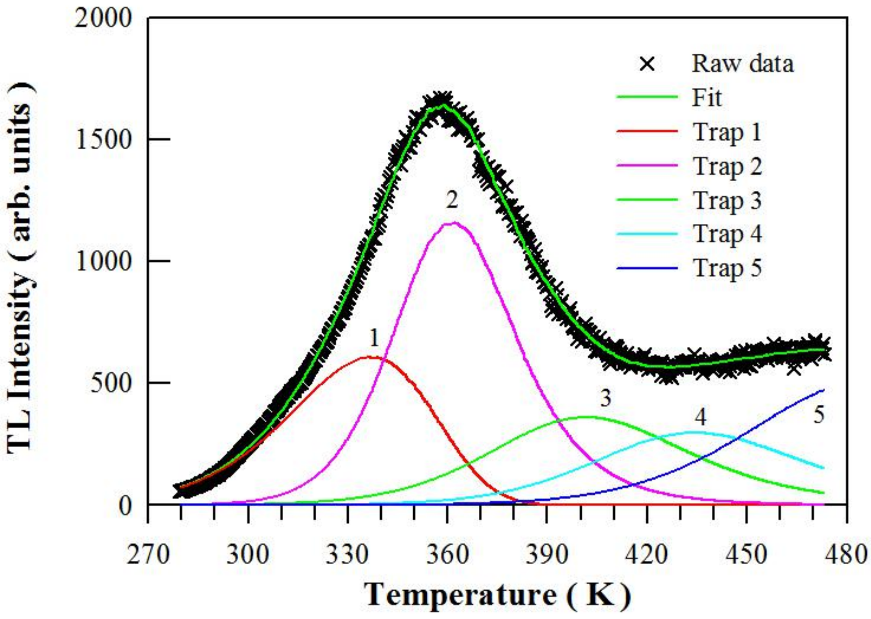

Figure S12. Computerized glow curve deconvolution of the TL glow curve of $CaAl_2O_4:Dy^{3+}$ (0.6 mol%) assuming the presence of 5 traps.

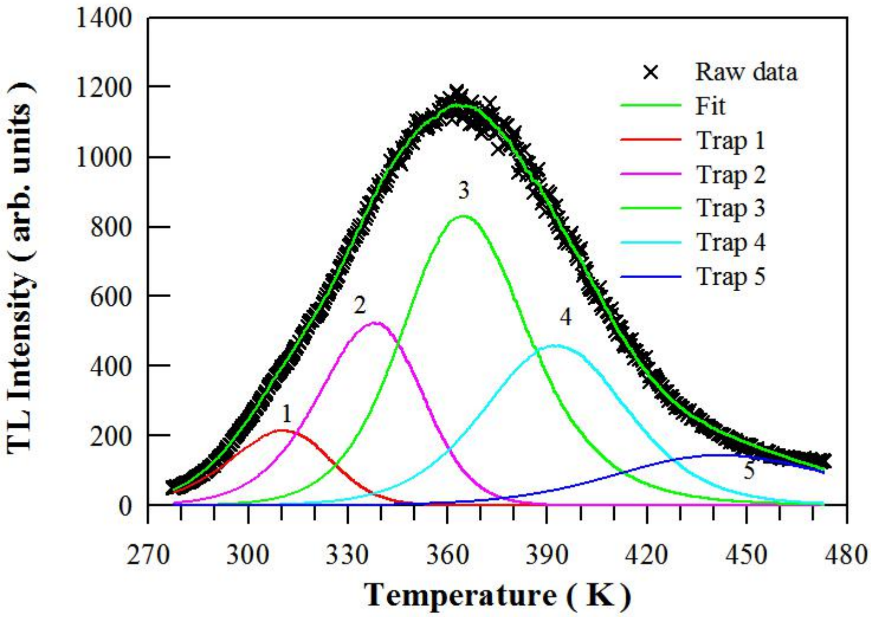

Figure S13. Computerized glow curve deconvolution of the TL glow curve of $CaAl_2O_4:Dy^{3+}$ (1.0 mol%) assuming the presence of 5 traps.

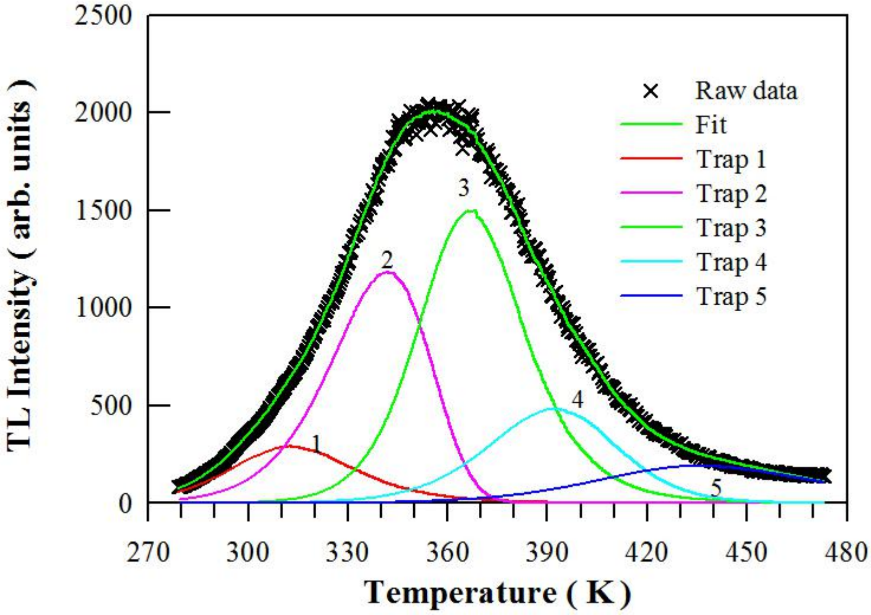

Figure S14. Computerized glow curve deconvolution of the TL glow curve of CaAl2O4:Dy3+ (2.0 mol%) assuming the presence of 5 traps.

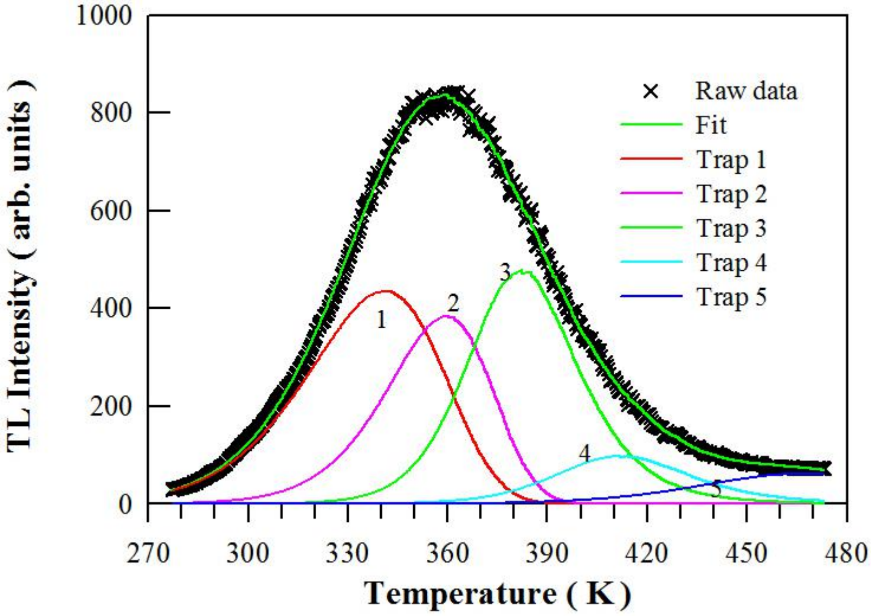

Figure S15. Computerized glow curve deconvolution of the TL glow curve of $CaAl_2O_4:Dy^{3+}$ (3.0 mol%) assuming the presence of 5 traps.

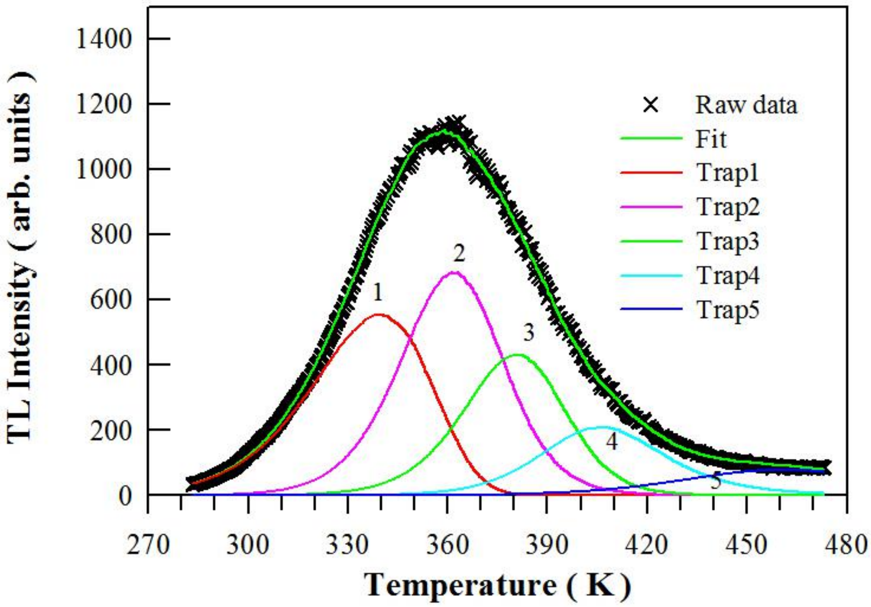

Figure S16. Computerized glow curve deconvolution of the TL glow curve of $CaAl_2O_4:Dy^{3+}$ (4.0 mol%) assuming the presence of 5 traps.

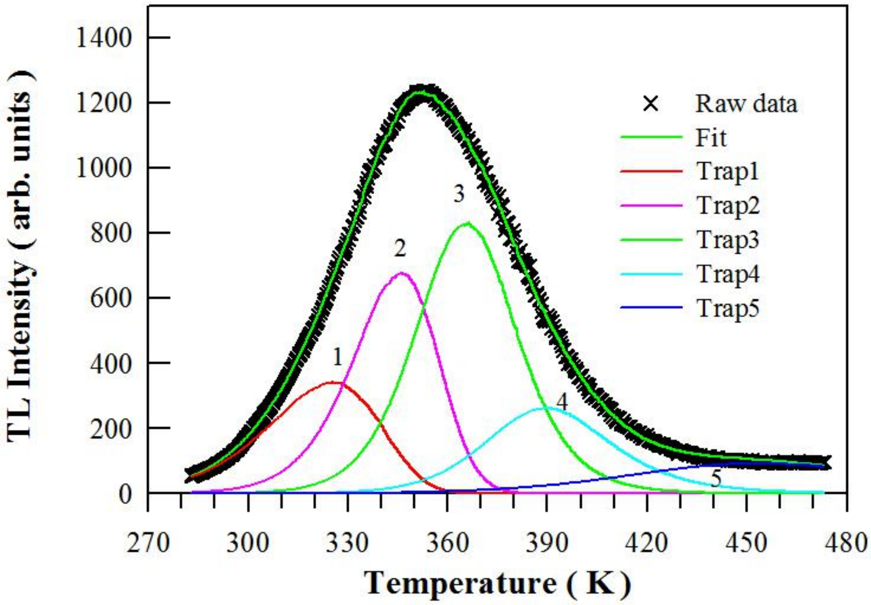

Figure S17. Computerized glow curve deconvolution of the TL glow curve of $CaAl_2O_4$:Dy^{3+} (5.0 mol%) assuming the presence of 5 traps.

Table S1. CIE chromaticity coordinates of $CaAl_2O_4:Dy^{3+}$ with different doping concentrations.

Doping Concentration (mol%)	*x*	*y*
0.1	0.156	0.094
0.2	0.160	0.112
0.4	0.161	0.112
0.6	0.162	0.127
0.8	0.165	0.116
1.0	0.165	0.131
2.0	0.170	0.140
3.0	0.173	0.151
4.0	0.175	0.156
5.0	0.187	0.176

Table S2. Kinetic parameters of the computerized glow curve deconvolution of the TL glow curve of $CaAl_2O_4:Dy^{3+}$ (0.1–5.0 mol%). Tm represents the peak temperature, E is the trap-depth, s is the frequency factor, b is the order of kinetics, and τ_{300} is the room temperature electron lifetime in the trap.

Dy^{3+} (mol%)	Trap Number	T_m (K)	E (eV)	*s* (s^{-1})	*b*	τ_{300} (h)	FOM (%)
0.1	1	308.45	0.5183	5.620×10^5	2.000	4.99×10^1	1.541
	2	346.05	0.5422	1.341×10^5	1.072	5.86×10^0	
	3	373.25	0.6626	1.429×10^6	2.000	6.30×10^3	
	4	45.45	0.5390	7.444×10^2	2.000	8.62×10^4	
0.2	1	308.75	0.5394	1.124×10^6	2.000	5.80×10^1	1.677
	2	349.15	0.4863	1.565×10^4	1.388	8.13×10^0	
	3	385.45	0.5739	4.797×10^4	1.738	2.06×10^2	
	4	468.15	0.5985	2.630×10^3	2.000	2.64×10^5	
0.4	1	305.95	0.5523	2.841×10^6	1.851	2.58×10^0	1.625
	2	352.15	0.5632	3.515×10^5	1.519	9.99×10^0	
	3	370.75	0.7134	9.680×10^6	1.403	1.19×10^2	
	4	401.25	0.7691	8.692×10^6	1.892	6.79×10^3	
	5	469.55	0.6885	2.866×10^4	2.000	8.85×10^5	
0.6	1	336.35	0.4218	2.949×10^3	1.000	2.00×10^0	2.477
	2	361.95	0.8471	1.524×10^9	2.000	9.47×10^3	
	3	380.65	0.8003	5.092×10^7	2.000	3.36×10^4	
	4	435.75	0.6944	1.450×10^5	2.000	2.22×10^5	
	5	470.00	0.7135	2.494×10^5	2.000	2.76×10^5	
1.0	1	309.55	0.6054	1.547×10^7	1.292	8.34×10^{-1}	1.845
	2	337.85	0.7103	9.232×10^7	1.350	1.01×10^1	
	3	365.45	0.8436	1.049×10^9	2.000	1.20×10^4	
	4	393.15	0.7714	1.471×10^7	1.653	1.37×10^3	
	5	441.15	0.6714	5.547×10^4	2.000	2.31×10^5	
2.0	1	312.55	0.6107	1.516×10^7	1.949	1.46×10^1	1.822
	2	341.75	0.6682	1.560×10^7	1.056	7.65×10^0	
	3	365.75	1.0271	3.679×10^{11}	2.000	5.25×10^4	
	4	391.85	0.8151	5.926×10^7	1.490	1.33×10^3	
	5	437.45	0.6661	6.219×10^4	2.000	1.67×10^5	
3.0	1	340.45	0.4635	1.095×10^4	1.000	2.86×10^0	1.793

	2	359.85	0.7015	1.427×10^{7}	1.065	3.20×10^{1}	
	3	382.15	1.0680	3.320×10^{11}	2.000	2.99×10^{5}	
	4	411.45	1.0064	4.489×10^{9}	2.000	1.88×10^{6}	
	5	467.85	0.7620	1.973×10^{5}	2.000	4.23×10^{6}	
4.0	1	338.25	0.5301	1.302×10^{5}	1.000	3.45×10^{0}	1.804
	2	362.85	0.8955	7.600×10^{9}	1.545	2.89×10^{2}	
	3	381.45	0.9801	2.385×10^{10}	1.472	2.34×10^{3}	
	4	407.35	1.0806	6.301×10^{10}	2.000	2.61×10^{6}	
	5	461.15	0.7808	4.522×10^{5}	2.000	2.25×10^{6}	
5.0	1	325.15	0.5154	1.824×10^{5}	1.000	1.37×10^{0}	1.352
	2	345.95	0.8433	5.237×10^{9}	1.156	2.81×10^{1}	
	3	366.05	1.0087	2.238×10^{11}	1.776	1.85×10^{3}	
	4	389.05	0.9748	9.669×10^{9}	2.000	2.45×10^{5}	
	5	453.05	0.5634	1.735×10^{3}	2.000	9.81×10^{4}	
